# Virtual reality for the assessment and rehabilitation of neglect: where are we now? A 6-year review update

**DOI:** 10.1007/s10055-022-00648-0

**Published:** 2022-05-30

**Authors:** S. Cavedoni, P. Cipresso, V. Mancuso, F. Bruni, E. Pedroli

**Affiliations:** 1 Applied Technology for Neuro-Psychology Lab, IRCCS Istituto Auxologico Italiano, Milan, Italy; 2 Department of Psychology, University of Turin, Via Verdi, 10, 10124 Turin, TO Italy; 3 Faculty of Psychology, eCampus University, Novedrate, Italy

**Keywords:** Virtual reality, Technology, Neglect, Assessment, Rehabilitation

## Abstract

Unilateral spatial neglect (USN) is a frequent repercussion of a cerebrovascular accident, typically a stroke. USN patients fail to orient their attention to the contralesional side to detect auditory, visual, and somatosensory stimuli, as well as to collect and purposely use this information. Traditional methods for USN assessment and rehabilitation include paper-and-pencil procedures, which address cognitive functions as isolated from other aspects of patients’ functioning within a real-life context. This might compromise the ecological validity of these procedures and limit their generalizability; moreover, USN evaluation and treatment currently lacks a gold standard. The field of technology has provided several promising tools that have been integrated within the clinical practice; over the years, a “first wave” has promoted computerized methods, which cannot provide an ecological and realistic environment and tasks. Thus, a “second wave” has fostered the implementation of virtual reality (VR) devices that, with different degrees of immersiveness, induce a sense of presence and allow patients to actively interact within the life-like setting. The present paper provides an updated, comprehensive picture of VR devices in the assessment and rehabilitation of USN, building on the review of Pedroli et al. (2015). The present paper analyzes the methodological and technological aspects of the studies selected, considering the issue of usability and ecological validity of virtual environments and tasks. Despite the technological advancement, the studies in this field lack methodological rigor as well as a proper evaluation of VR usability and should improve the ecological validity of VR-based assessment and rehabilitation of USN.

## Introduction

A cerebrovascular accident, such as stroke, represents an urgent public health issue and patients surviving this catastrophic acute event must deal with life-long motor and cognitive disabilities. This could restrict patients’ participation to social activities, providing them and their caregivers a relevant psychological burden (Chen et al., 2013; Béjot et al., 2016; Mansfield et al., 2018). Depending on location, type, and severity of the cerebrovascular occlusion, patients typically exhibit two main categories of impairments following the acute phase of a stroke: (i) motor disability, manifested with the inability to walk, problematic coordination and balance, hemiplegia or hemiparesis; (ii) cognitive and neuropsychological impairments, including aphasia, amnesia, executive dysfunctions, apraxia, impaired visuospatial abilities, and mood disorders (Sundar and Adwan 2010; Chen et al., 2013; Sun et al., 2014; Pedroli et al., 2015; Jokinen et al., 2015; Cipresso et al., 2018a). Approximately, 50% of patients who suffered from right-brain stroke manifest unilateral spatial neglect (USN), a complex and heterogeneous attentional-perceptual syndrome characterized by a difficulty or inability to pay attention, detect, and orient toward stimuli presented in the contralesional side (Heilman, et al., 2000; Tsirlin et al., 2009; Pedroli et al., 2015; Rode et al., 2017; Zigiotto et al., 2020). USN can be divided into several subcategories, depending on whether the behavior is elicited by a sensory, motor, or representational modality, or whether it involves one’s peripersonal, extra-personal, or spatial representation (Plummer et al., 2003; Buxbaum et al., 2004; Grattan and Woodbury, 2017). Thus, USN can also be referred to as visuospatial neglect (VSN), visual neglect (VN), and hemispatial neglect (HSN). For the purpose of this review, we will consider the terms USN and VSN interchangeably. Approximately, 50% of patients manifest USN following a stroke concerning the inferior parietal lobe, the superior temporal lobe, the frontal cortex, and subcortical nuclei. Moreover, a right-brain damage accounts for 90% of USN patients (Buxbaum et al., 2004; Yasuda et al. 2017, 2018). Studies that employed functional magnetic resonance imaging (fMRI) on VSN patients’ brains have also shown lesions in the right superior and medio-temporal gyri, the basal ganglia, as well as white matter tracts damages in both uncinate fasciculus and inferior occipitofrontal (Karnath et al., 2011; Vuilleumier, 2013; Lunven et al., 2015; Wahlin et al., 2019). Furthermore, the deficits include the ventral and dorsal areas of the attention networks, placed in the fronto-parietal portion of the brain; the ventral attention network (VAN) includes the temporo-parietal and inferior-frontal right cortex and accounts for the detection of unexpected, relevant stimuli, whereas the dorsal attention network (DAN) accounts for the top-down selection of stimuli and comprises portions of the intraparietal and superior frontal cortex (Corbetta et al., 2005; Ogourtsova et al., 2018a, 2018b; Wahlin et al. 2019; Zigiotto et al., 2020).

At a behavioral level, USN can manifest when patients rotate their head and eyes to the impaired side and focus their attention on the central, unaffected visual field to register information (thus manifesting visuospatial recognition impairment and visual field defects, VFD; Sugihara et al., 2016). USN patients fail to orient their attention and detect contralesional auditory, visual, and somatosensory stimuli, as well as to collect and purposely use information located in their contralesional side (Booth, 1982; Heilman et al., 2000; Tsirlin et al., 2009; Aravind and Lamontagne 2018; Wahlin et al. 2019; Zigiotto et al., 2020). Moreover, patients affected by USN can collide with static or moving people or objects placed on the contralesional near or far space while performing tasks (Tsirlin et al., 2009; Aravind and Lamontagne, 2014, 2018; Aravind et al., 2015). The higher collision rates seem related to a delay in detecting obstacles and adopting an adequate strategy to avoid them (Aravind and Lamontagne, 2014, 2018; Aravind et al., 2015). Patients’ impaired judgment of distances from objects could also reflect an ipsilesional shift of their subjective midline, which acts as a framework for goal-directed walking and spatial orientation (Karnath et al., 1991; Richard et al., 2004; Aravind and Lamontagne, 2018). USN patients also tend to miss words while reading (Kim et al., 2015; Sugihara et al., 2016; Yasuda et al., 2017, 2018), and present a reduced ability to manage both basic and instrumental activities of daily living (BADL and IADL, respectively), such as autonomously bathing or grocery shopping (Buxbaum et al., 2004; Grattan and Woodbury, 2017). Furthermore, USN patients manifest different degrees of unawareness of their impairments (i.e., anosognosia) and this condition is associated with motor, cognitive, and sensory deficits. This could result in longer rehabilitation periods, the need for a constant supervision and, overall, poorer prognosis along with a reduced possibility to benefit from rehabilitative interventions (Cherney et al., 2001; Azouvi et al., 2003; Di Monaco et al., 2011; Nijboer et al., 2008, 2014; Tobler-Ammann 2017a, 2017b; Glize et al., 2017; Cipresso et al., 2018a; Zigiotto et al., 2020). This entails the need for a prompt assessment and rehabilitation of USN, in order to reduce its detrimental effects over patients’ functioning and quality of life.

## Current USN assessment and rehabilitation

### USN assessment

Among the numerous paper-and-pencil standard neuropsychological assessment tools for USN, the most frequent are: (i) cancellation tests, that require patients to detect specific targets among numerous other distractors, employing stimuli such as lines (Albert, 1973), letters (Diller and Weinberg, 1977), symbols (Weintraub and Mesulam, 1988), and circles (Vallar and Perani, 1986; Pallavicini et al., 2015a; Pedroli et al., 2015); (ii) copy tests, where patients are asked to copy a simple or complex picture or reproduce it from memory (Rey, 1941; Suhr et al., 1998; Shulman 2000); (iii) line bisection tests, which require patients to draw the midline of horizontal lines (Schenkenberg et al., 1980; Pallavicini et al., 2015a; Sugihara et al., 2016). Other measures employ a more ecological approach to USN assessment: (iv) the Behavioral Inattention Test (BIT, Wilson et al., 1987, 2010), a short screening battery of tests to assess the presence and the severity of neglect symptoms in everyday skills. Moreover, some of the tests included could be compared or used separately for a qualitative description of the patient’s functional performance (Hartman-Maeir and Katz, 1995), despite a similar proposal is currently hypothetical and lacks a proper validation; (v) the Catherine Bergego Scale (CBS; Bergego et al., 1995; Azouvi 1996), a standardized checklist used to detect the presence and degree of USN symptoms during the observation of patients performing in daily situations. It also compares patients’ and caregivers’ evaluations to verify the presence of anosognosia (Azouvi et al., 2003; Sugihara et al., 2016). These measures could be considered more ecological because they address patients’ functional performance and their ability to autonomously perform daily life activities, which could be useful for a more sensitive detection of milder forms of USN (Ogourtsova et al., 2018b). In fact, traditional paper-and-pencil tests do not appear sufficiently sensitive in detecting subtle impairments which, over time, could have a detrimental effect and worsen patients’ functioning and life quality (Bowen et al., 1999; Buxbaum et al., 2008; Aravind and Lamontagne, 2014; Ogourtsova et al., 2019, 2018b). These tests, and particularly the line bisection, generally require a manual correction by an experienced clinician; this could increase the risk of biases, inter- and intra-rater variability (Jee et al., 2015). Moreover, clinical assessment of USN is usually limited to near peripersonal space (within arm’s reach) and fails to consider other behavioral manifestations in personal and motor symptoms along with far extra-personal space (beyond the arm’s reach) (Azouvi et al., 2003; Ogourtsova et al., 2018b; Knobel et al., 2020) and egocentric versus allocentric spatial representations as well (Bickerton et al., 2011; Pedroli et al., 2015; Ogourtsova et al., 2018b).

### USN rehabilitation

Rehabilitative interventions for USN patients usually fall under two broad categories of behavioral approaches (Pedroli et al., 2015; Azouvi et al., 2017; Rode et al., 2017; De Luca et al., 2019; Liu et al., 2019; Zigiotto et al., 2020): (i) top-down, activity-based approaches aim at orienting patients’ spatial attention toward the left side of space and to promote their functional abilities. Over the past 40 years, several systematic visual scanning training (VST) programs have been developed (e.g., Pizzamiglio et al., 1992; Antonucci et al., 1995) to stimulate patients to actively explore their contralesional (neglected) side, with therapists asking them to voluntarily direct their gaze leftward (Tsirlin et al., 2009; Liu et al., 2019; Zigiotto et al., 2020). The visual search includes tasks such as scanning, copying, or reading stimuli placed in the contralesional side of space and can be guided either by contralesional cues or therapists’ feedback. Other top-down approaches include the limb activation treatment (LAT), in which patients are asked to perform intentional movements using their contralesional hemibody (Rizzolatti and Berti, 1990; Robertson and North, 1992, 1993) (ii) bottom-up, non-activity-based interventions; these latter methods aim at reducing patients’ bodily deficits using external instruments to manipulate the sensory environment. This kind of intervention also exploit physical stimulation and manipulate patients’ sensory environment to improve neglect symptoms with methods such as hemiblinding, eye-blinding, caloric, galvanic or optokinetic stimulation (OKS; Pizzamiglio et al., 1990; Robertson et al., 1998; Moon et al., 2006; Kim et al., 2015; Azouvi et al., 2017), or prism adaptation (PA; Tsirlin et al., 2009; Jacquin-Courtois et al., 2010, 2013; Azouvi et al., 2017; Glize et al., 2019; Liu et al., 2019). OKS has proved particularly effective for treating left hemispatial neglect; by activating brain stem, basal ganglia, cerebellum, and the parieto-occipital cortex, it improved distorted body orientation, tactile extinction, motor neglect, and even better attention to auditory stimuli. This is particularly true when OKS include leftward moving stimuli such as dots (Vallar et al., 1993, 1997), vertical strip or random dot backgrounds (Kim et al., 2007), or drums (Moon et al., 2006), whereas rightward OKS appear to worsen left hemispatial neglect (Kim et al., 2015). PA, instead, consists of actively exposing patients to a rightward optical deviation of their visual field, with the aim to reorient their behavior toward the neglected side. To achieve this, the procedure exploits prisms that systematically shift both visuomotor and proprioceptive responses to the left (Jacquin-Courtois et al., 2010, 2013; Azouvi et al., 2017; Glize et al., 2019). The process of PA requires patients to repeatedly perform movements toward visual targets placed in the contralesional side, with prisms deviating the environment about 10° rightward. The prismatic exposure is preceded by a pre-test phase, in which patients aim at the direction of visual targets in order to obtain reference values, without wearing glasses. Following prismatic exposure, patients are asked to aim toward the visual targets without the prisms, in order to evaluate the after-effects (Azouvi et al., 2017).

OKS and PA have received increasingly more interest over the years; on the one hand, OKS involves USN patients to observe moving visual targets, to encourage their visual scanning of the neglected hemispace. The initial research conducted on OKS showed that the exposure to a moving stimulus, which included optokinetic nystagmus, could modify patients’ analysis of the perceived space (Pizzamiglio et al., 1990). On the other hand, PA has proved as one of the most effective and widely employed rehabilitation methods which reduce the behavioral biases and the awareness deficits seen in the contralateral hemispace of spatial neglect. The active prism exposure allows to re-calibrate attention and re-orients patients’ behavior toward the neglected side, reducing visual, sensory, and auditory neglect (Dijkerman et al., 2003; Jacquin-Courtois et al., 2010), space and object-based neglect (Dijkerman et al., 2003; Maravita et al., 2003), and spatial dyslexia and dysgraphia (Farnè et al., 2002; Rode et al., 2006). Mental imagery and higher-level spatial representations seem to benefit from PA as well; however, it is unclear whether this impact can be broadened to navigation and topographic memory, which contribute to spatial cognition (Glize et al., 2017). Therefore, despite the positive outcomes following the traditional PA rehabilitation, to date, its mechanisms are largely unclear.

## What is virtual reality?

A major drawback of a traditional approach to USN is that cognitive functions are considered as a variable isolated from the actual context patients live in. This could hinder the ecological validity of both assessment and rehabilitation processes; in other words, the observations and data obtained from the traditional methods could not be generalized in order to understand how patients function in their real-life surroundings. This entails methodological as well as practical issues that could be improved by using tools that allow simulating patients’ response to stimuli or situations that closely resemble the ones they would encounter outside the clinical setting (Tsirlin et al., 2009; Levick, 2010; Pallavicini et al., 2015a; Ogourtsova et al., 2019, 2018a, 2018b). A plausible solution comes from integrating standard procedures with new technologies, such as virtual reality (VR). VR is a 3D computer-generated environment that allows to virtually recreate real-life contexts in which the individual can feel immersed and “present” and can interact with the surroundings (Riva and Mantovani, 2014, 2019; Riva et al., 2018; Moreno et al., 2019). The interaction with the virtual environment provides the individual with immediate multisensorial feedback (e.g., visual, haptic, auditory), and is highly responsive to the users’ movements and inputs (e.g., gesture, vocal command) (Burdea and Coiffet, 2003; Riva, 2008). Moreover, the concurrent stimulation of multiple senses and the reality’s closeness of the stimuli employed can induce the feeling of *immersion* within a safe and controlled VR environment and the possibility to *interact* with objects (Rizzo et al., 2004; Riva, 2008; Slater, 2009; Riva and Mantovani, 2012; Chirico et al., 2016; Riva et al., 2018; Cipresso et al., 2018b). Several VR devices have been developed over time and, depending on their degree of immersiveness, can be categorized in: (i) non-immersive systems, such as computer screens; (ii) semi-immersive systems, such as the cave automated virtual environment (CAVE; Cruz-Neira et al., 1993) which makes use of large, fixed screens distant from the viewer available in many configurations. The three-wall setup provides a 180° scenario that can be considered semi-immersive because as soon as the patient turn over he or she is no longer immersed in the virtual environment; (iii) fully immersive systems, such as head-mounted displays (HMDs) and four-wall setup of the CAVE.

Specifically, the fully immersive devices are capable of isolating individuals from the external “real” environment and, most importantly, generate the feeling of *presence,* i.e., the feeling of being really “there” in the simulated environment and being able to act purposively within it (Rizzo et al., 2004; Slater, 2009; Riva and Mantovani, 2012, 2014; Negut et al., 2016; Chirico et al., 2016; Riva et al., 2018). The integration between the input data, collected via trackers sensing the user’s position and orientation, and a real-time update of the virtual environment generates a high sense of presence (Riva et al., 2018). This allows achieving a brain activity like that spontaneously elicited when the individual interacts with the real-life surroundings and predicts the inputs incoming from the context in which he is immersed. A more detailed examination of these aspects will be presented later in this paper and will consider a plausible explanation for VR effectiveness based on the concept of the “body matrix” (Moseley et al., 2012; Riva, 2018).

### Technological advancements in USN assessment and rehabilitation

Over time, VR has been extensively applied within the field of neuropsychological assessment and rehabilitation of clinical and non-clinical populations of young adults and elderlies (García-Betances et al., 2015; De Tommaso et al., 2016; Plancher and Piolino, 2017; Riva et al., 2020). Clinical subsamples include, among others, patients suffering from spatial memory (Allain et al., 2014), balance impairments (Serino et al., 2017a, bGerber et al., 2018; Soares et al., 2018), and from the consequences of stroke (Henderson et al., 2007; Saposnik and Levin, 2011; Laver et al., 2017), traumatic brain injury (Aida et al., 2018; Alashram et al., 2019; Maggio et al., 2019), and neurocognitive disorders (Moreno et al., 2019).

**Fig. 1:**
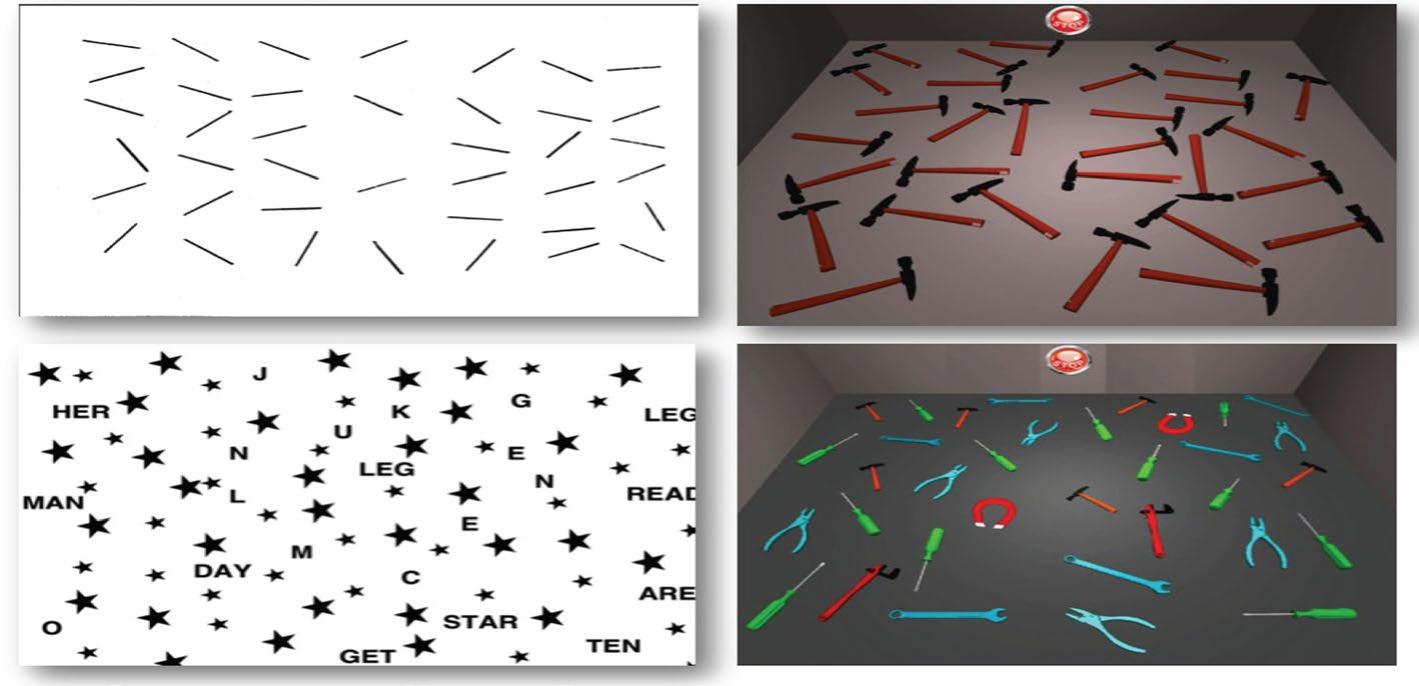
Paper-and-pencil versus computerized USN assessment (courtesy of Pallavicini et al., 2015b)

Specifically, both USN assessment and rehabilitation seemingly share common issues: despite the vast amount of measures and interventions, a gold standard procedure is currently lacking (Bowen et al., 1999; Menon and Korner-Bitensky, 2004; Pedroli et al., 2015; Grattan and Woodbury, 2017; Ogourtsova et al., 2019, 2018b). USN presents with a complex and heterogeneous set of manifestations and no assessment tool nor rehabilitative training alone could comprehensively address this condition (Menon and Korner-Bitensky 2004; Pedroli et al., 2015; Ogourtsova et al., 2019, 2018b). Furthermore, both employ measures and tasks which often lack sensitivity, standardization, and ecological validity, thus fail to evaluate how patients perform relevant tasks in their daily environment (Perez-Garcia et al., 1998; Azouvi et al., 2003; Tsirlin et al., 2009; Levick, 2010; Pallavicini et al., 2015a; Ogourtsova et al., 2019, 2018a, 2018b). Regardless of the method used, early assessment and prompt rehabilitation are crucial for USN patients and could improve their behavioral and socio-cognitive outcomes: this, in turn, would reflect a decreased hospitalization rate and reduced healthcare assistance costs. Promoting at-home interventions is also crucial for outpatients to maintain and consolidate the positive outcomes following the rehabilitation training, even after dismissal Mugueta-Aguinaga and Garcia-Zapirain 2017; Serino et al. 2017a, 2017b; Kidd et al., 2019). Over time, these limitations have led many researchers to transfer conventional paper-and-pencil assessment tools and rehabilitative training in a computerized form. This transition has represented a first step forward in enhancing both precision and consistency in detecting more subtle forms of deficits and in recording patients’ performance (Tsirlin et al., 2009; Pallavicini et al., 2015a; Pedroli et al., 2015). A computerized task differs from a paper-and-pencil task not only for the format in which the stimuli are presented (i.e., a monitor instead of a sheet), but also for the cognitive and motor demands required to perform the task itself (Jee et al., 2015). Figure 1 illustrated a computerized version of the card dealing task and the barrage task. Paper-and-pencil tasks recruit patients’ motor abilities and visuospatial attention, whereas computerized tasks increase perceptual sensitivity while reducing the motor action required (Jee et al., 2015). The attentional capacity of USN patients tends to reduce as a result of increasingly demanding perceptual tasks; this, in turn, reduces patients’ ability to sustain and orient their attention to the ipsilesional side. Computerized tasks seemingly reduce the motor and attentional load required (Jee et al., 2015). Furthermore, computer-based assessment and rehabilitation provide information unobtainable from the traditional assessment, such as reaction times.

**Fig. 2:**
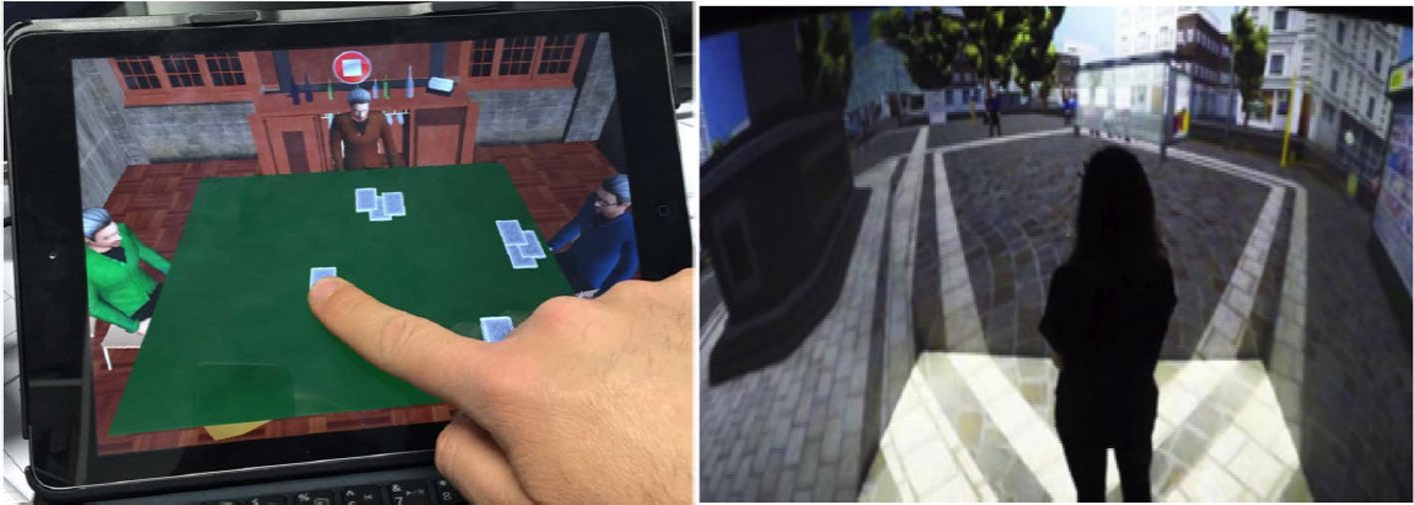
New technologies for USN assessment and rehabilitation: an iPad (on the left; courtesy of Pallavicini et al., 2015a) and a 4-wall CAVE (on the right; courtesy of Riva 2022)

However, despite their utility, computerized systems are still not capable of simulating a complex set of actions and do not provide an ecological environment with a high sense of realism and presence. This also limits patients’ possibility to interact with the device providing the stimuli. Therefore, the most recent technological advancements within the field of VR have allowed a second major transition, from non-immersive toward fully immersive devices (e.g., HMDs, CAVE) capable of providing a safe, standardized, and more realistic virtual environment that simulates complex daily situations, directly involving patients and engaging them to purposely interact within it (Slater, 2009; Cipresso et al., 2018b). These crucial properties have fostered the widespread diffusion of VR for the assessment and rehabilitation of many neurocognitive disorders (Rizzo et al., 2004; Parsons et al., 2013; García-Betances et al. 2015; Negu et al., 2016; Aida et al., 2018; Cipresso et al., 2018b; Alashram et al., 2019; Moreno et al., 2019; Kim et al., 2019; Maggio et al., 2019; Riva et al., 2020) (Fig. 2). VR-generated environments allow a strict experimental control over stimulus administration and either the assessment or the rehabilitation procedure can be adjusted and tailored to patients’ needs and difficulties and carried out safely. VR-based setups also allow examining not only the peripersonal space, but also the extra-personal space (Knobel et al., 2020). Furthermore, clinicians could benefit from the employment of VR in many ways; this technology could help them detect subtle manifestations of deficits, which could remain unrecognized and have a detrimental effect on patients over time. In terms of assessment, this would be fostered using virtual environments that examine patients’ functioning and impairments. This technology also allows to gradually increase the complexity of the tasks required, which could provide a refined assessment of patients’ impairments. A VR-based rehabilitation, instead, could gradually train patients to carry out tasks similar to those they perform in their daily life and could learn again to execute them in a safe and controlled environment. This also rules out possible interfering variables and enables to suspend the procedure whenever needed; despite VR having very few side effects, sometimes patients can manifest cybersickness (CS), a form of visually induced motion sickness producing a constellation of symptoms and discomfort during or following VR exposure (Martirosov and Kopecek, 2017; Weech et al., 2019; Knobel et al., 2020). CS symptoms include disorientation, nausea, headache, fatigue, and postural instability, due to the mismatch between sensory inputs, since the user sees the movement on the screen without feeling it (Martirosov and Kopecek 2017).

Given the importance of VR devices for assessment and rehabilitation, particularly within the field of USN, a review of the most recent technological advancements is needed to have a comprehensive state-of-the-art and to provide future directions for researchers. Therefore, the present review aims at updating the previous work of Pedroli et al. (2015) and to present the most recent evidence of VR advancements for both assessment and rehabilitation of USN. Specifically, this article will review VR applications from the years 2015 to 2021 included, analyzing the methodology and the technologies employed in the studies analyzed in order to understand the advancements in this field and future research directions. The following sections will consider how VR technologies have been implemented within standard neuropsychological assessment and rehabilitation; it is crucial to remind that VR does not intend to replace traditional methods, but rather to enhance them and help increase the amount of data collected to provide a fuller, more comprehensive and ecological picture of patients’ impairments and help to tailor safe interventions closer to their needs.

## Methods

The present review builds on and updates the previous work by Pedroli et al. (2015), following the Preferred Reporting Items for Systematic Reviews and Meta-Analysis (PRISMA) guidelines (Moher et al., 2009). This section will present the systematic strategy and the criteria employed to select the studies, which will be discussed later.

### Search Strategy

A computer-based search strategy for relevant publications was performed considering several databases, specifically PsycINFO, Web of Science (Web of Knowledge), PubMed/Medline. In line with the previous work of Pedroli et al. (2015), the search string employed was (“Virtual Reality” OR “Technolog*”) AND [“Neglect” OR (“Unilateral Spatial Neglect” OR “Hemispatial Neglect” OR “Visual Neglect” OR “Visuospatial Neglect”)]. The choice to include both “Virtual Reality” and “Technolog*” as keywords were to avoid possibly missing papers since these words are sometimes used in an interchangeable or misleading way. We also wanted this review to be as replicable and inclusive as possible. The articles were individually considered to verify whether they fulfilled the following inclusion criteria: (a) research article; (b) provide information regarding the sample used; (c) provide information regarding the measures used; (d) published in English. We excluded conference papers; articles that did not consider neglect as a neuropsychological syndrome (e.g., childhood traumatic experiences, maltreatment, and abuse).

### Systematic Review Flow

**Fig. 3:**
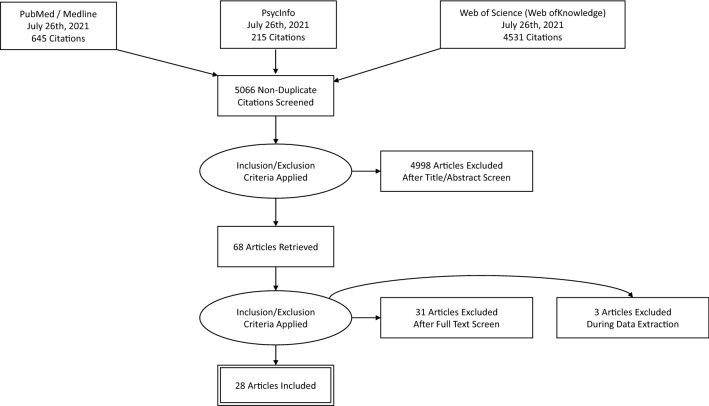
Search strategy and flow diagram for the database search of the systematic review

The flowchart of the review is shown in Fig. 3. By searching in PubMed/Medline, Web of Science (Web of Knowledge), and PsycINFO, our initial search yielded a total number of 5066 non-duplicate citations. Considering our inclusion and exclusion criteria, we retrieved 68 articles that were further screened. After the full-text screening, we selected 31 articles, and two additional full papers were excluded in the data extraction phase. In the end, 28 studies met the full criteria and were included in the present review. The risk of bias was assessed using PRISMA recommendations for systematic literature analysis. SC, PC, and EP independently selected paper titles and abstracts and analyzed the full papers meeting the inclusion criteria. Any disagreement was resolved through consensus. SC wrote the manuscript, and all the authors (PC, EP, VM, and FB) read, revised, and approved its final version.

## Results

### VR systems for USN assessment

The field of USN assessment could benefit from more ecological devices capable of detecting this condition more precisely, to overcome the lack of sensitivity, specificity, and ecological validity of paper-and-pencil tests. Over the past five years, several studies have integrated VR-based devices in USN assessment, either digitalizing traditional neuropsychological tests or developing new paradigms. A total of 15 studies was selected; six studies employed non-immersive technologies (by means of a tablet/iPad, computer screen, and auditory stimulation: Pallavicini et al., 2015a; Jee et al., 2015; Guilbert et al., 2016; Grattan and Woodbury, 2017; Spreij et al., 2020; Siddique et al., 2021); nine studies employed fully immersive technologies (by means of HMDs: Aravind et al., 2015; Sugihara et al., 2016; Aravind and Lamontagne, 2017, 2018; Ogourtsova et al., 2018a, 2018b; Knobel et al., 2020; Yasuda et al., 2020; Kim et al., 2021), which will be illustrated as it follows. Table 1 provides a detailed description of the VR technology employed in the following studies, as well as the characteristics of the sample, the sessions, and the main outcomes.

**Table 1:** Technological advancements in neglect assessment. The table briefly summarizes (a) sample size and description; (b) features of the VR device employed; (c) the conditions and tasks of the study; (d) main outcomes

VR AND TECHNOLOG* FOR NEGLECT ASSESSMENT				
References	Characteristics of sample	Characteristic of VR applications	Sessions	Main outcomes
Aravind et al., 2015	Post-stroke VSN patients (n = 12, aged 47–72; 8 females, 4 males)	The virtual environment was viewed in a nVisor SX60 head-mounted display (HMD) (NVIS) and was controlled with CAREN-3™ virtual reality software (Motek BV). Patients responded using a joystick (Attack3- Logitech) with their non-paretic hand	The session included a clinical assessment (VSN- Bell test; Line Bisection test; MoCA; TMT-B; Edinburgh Handedness Inventory) and three laboratory tasks: (i) obstacle detection task; (ii) joystick-driven obstacle avoidance task; (iii) locomotor obstacle avoidance task. Practice trials were provided before the actual assessment (ten trials for each of the four conditions)	VSN is specifically involved in the altered behavior of obstacle avoidance. Patients displayed a precise pattern of collision, and also a correlation between the distance at the time of detection and at the onset of the avoiding strategy. Moreover, VSN patients displayed specific avoidance strategies consisting of both changing mediolateral deviating and increasing speed
Pallavicini et al., 2015a	Sixteen patients with right-hemisphere damage due to cerebrovascular lesion, without hemianopia, divided into two groups: with USN (five males, three females) and without USN (7 males, 1 female). Group mean age: 66.1 ± 11.9	Neglect App, the iPad application developed for USN evaluation. Includes two task categories: Neglect App cancellation tests (simple and with distractors); Neglect App card dealing task. These tasks recreate the paper-and-pencil version of the cancellation test and the Card Dealing task	Patients completed a preliminary clinical interview and a neuropsychological evaluation (MMSE). Within a single testing session, participants completed a technological skills assessment questionnaire. Participants were randomly asked to complete the paper-and-pencil and Neglect App test, in a randomized order. A ten-minute training period was included before the Neglect App. After the Neglect App test, they completed a SUS questionnaire	The Neglect App cancellation tests proven itself as equally effective to the traditional paper-and-pencil tests in screening symptoms between patients with and without neglect. The Neglect App Card Dealing task showed more sensitivity in detecting neglect symptoms than a traditional functional task. For the cancellation task, USN patients showed aberrant search performance in both paper-and-pencil and Neglect App tests. For the Card Dealing task, USN patients reported higher omissions in the Neglect App but not in the paper-and-pencil test
Jee et al., 2015	Patients with unilateral visual neglect (ULN; n = 11, 10 males, 1 female; mean age = 63.54 ± 14.72). The e-system was firstly tested on a group of healthy participants (n = 42, 21 males, 22 females: mean age = 24.65 ± 2.87) and on a second cognitively healthy group after a recalibration of the e-system (n = 10, 5 males, 5 females; mean age = 23.6 ± 2.18)	The e-system included an electronic pen (e-pen), a micro-pattern printed paper and a line bisection test software, installed on a computer. The e-pen detects and sends written/drawn information from the micro-pattern printed paper to the LBT software, and it consists of a pressure sensor, an infrared LED, image sensor, a digital signal processor and a Bluetooth interface	The session required patients to use their right hand to cut each line in half, by placing a small pen notch in the midline of each stimulus. Patients were asked to mark only one pen notch without skipping any line, and to not move the test page	The e-system is evaluated as feasible on cognitive health subjects; in comparison with the traditional paper-and-pencil tests, the semi-computerized system proved to be both valid and reliable in assessing ULN patients. Moreover, assessing and recording test results automatically, it significantly reduced assessment and recording time, resulting in increased efficiency
Guilbert et al., 2016	The first experiment enrolled only healthy participants. The second experiment involved a group of 14 patients total, with USN (n = 10, 5 in each task –detection and lateralization) and without USN (n = 4; 2 in each task –detection and lateralization)	The 3D virtual environment was created using the SLAB Audio-Spatial Renderer, with custom-made software running on a laptop. The auditory stimuli were presented by means of Sennheiser HD 165 headphones. Patients were blinded to avoid visual interferences and had to provide their response using a mouse placed in front of them	Two auditory tasks were used, detection and lateralization. In the detection task, patients had to press a mouse button as soon as they heard the target, a complex harmonic sound. The lateralization task required patients to press either the left or right mouse button following the spatial position of the target. Catch trials (cues in absence of targets) were also present	For the detection task, no evidence of recruiting spatial processes for sound detection was found for both patients, with or without USN, and healthy controls. Moreover, no evidence was found for significant cueing effects. For the lateralization task, USN patients appear to manifest difficulties in auditory orienting, and spatial cueing appeared useful when a spatial judgment was required to locate the target
Sugihara et al. 2016	Eight patients, divided into two groups: USN (n = 4, aged 26–79, one female, three males), VFD (n = 4, aged 36–60, one female, three males)	HMD system displays the stimuli on an LCD screen. Two miniature CMOS cameras were installed in the HMD to detect each eye’s movement, analyzed by means of the Frame-DIAS IV software	Patients sitting on a chair firstly completed a line cancellation test without the HMD. Then, patients performed the HMD line cancellation test under four conditions: no reduction in the test sheet image; center reduction of the sheet; left and right reductions of the sheet. The left and right sides of the test sheet were blanked alternatively	VFD patients performed correctly under any condition of the left and right test sheets displayed on the HMD. USN patients’ answer rates, instead, performed correctly in the left paper-and-pencil condition. Their performance dropped significantly in the HMD conditions (either no-reduction, center, right and left reduction)
Aravind & Lamontagne (2017)	A total of 26 patients following a right-sided stroke were divided in two groups: VSN + (n = 13; mean age = 59.8 ± 7.7) and VSN- (n = 13; mean age = 60.8 ± 6.5)	A virtual environment was created by means of the CAREN3 software (MotekBV). Patients viewed the virtual environment within a nVisor SX60 head-mounted display (HMD) apparatus (NVIS); on the HMD were affixed 3 reflective markers and a 12-camera Vicon-512 motion capture system was used	Patients performed: (i) Cognitive Single Task (CogST), an auditory discrimination pitch task (in a simple and complex version) while seated and observing the virtual environment. (ii) Locomotor Dual task (LocoDT), involving obstacle avoidance while performing the simple and complex cognitive tasks	VSN + patients display a greater deterioration in locomotor and cognitive performances while dual tasking, as well as a greater collision rates with delays in obstacle perception. Dual-task walking and increased task complexity have a significant detrimental effect on both cognitive and locomotor performances of VSN patients and is associated with delayed patients' initiation of an avoiding strategy and greater locomotory costs, possibly due to deficits in executive functions
Grattan & Woodbury, 2017	Twelve participants (six males, six females) aged between 52 and 79 years. Eleven showed cognitive impairment and one had hemianopsia. Six participants were outpatients and 6 were inpatients	Participants completed the Enhanced array of Virtual Reality Lateralized Attention Test (VRLAT), running on a PC laptop using a Logitech Extreme 3D Pro joystick. This version provides patients with multiple distractors (i.e., objects, auditory and moving)	VRLAT was compared to functional, paper-and-pencil and descriptive assessments. Patients travel down a virtual path and are asked to identify targets placed on both left and right sides of the screen. One or two sessions were required depending on patients’ availability	Functional and VR assessment measures outperform paper-and-pencil tests in detecting neglect
Aravind & Lamontagne (2018)	A total of 26 patients with right-hemisphere stroke participated and were divided in two groups: VSN + (n = 13, mean age = 59.8 ± 7.7) and VSN- (n = 13, mean age = 60.8 ± 6.5). A small control group of healthy subjects was included as well (n = 5, aged 50–74 years, three males and two females)	An nVisor SX60 head-mounted display (HMD) apparatus (NVIS); a 12-camera Vicon-512 motion capture system tracked the position of 3 reflective markers fixed to the HMD; the CAREN 3 virtual reality software (Motek BV); reflective markers placed on specific body landmark and on the walking aid when possible. Patients also used a joystick (Attack3- Logitech for the perceptual task	Patients performed: (i) a locomotor obstacle task, where they had to walk toward the target and simultaneously avoid the collision with an obstacle approaching; (ii) a perceptual task in a seated position, where they pressed a joystick button as soon as they detected a moving obstacle. Patients underwent a clinical assessment as well (including VSN: Bells Test; Apples test; Line Bisection test; MoCA; TBT-B; Activity-specific Balance Confidence scale, ABC)	VSN- and HC avoided obstacles with a specific strategy, deviating to the same side as the obstacle or to its opposite side, minimizing the risk of colliding. Compared to VSN- and HC, VSN + patients showed higher collision rates with contralesional static and dynamic objects; attentional/perceptual bias resulting in more time spent with their head oriented toward the ipsilesional side and their tendency to rightward deviate their path while walking; poorer variability in locomotor responses, executive functions and ability to reorient toward the goal
Ogourtsova et al. 2018a	Thirty patients divided into two groups: with USN (USN + ; n = 15; mean age = 60.2 ± 8.8 years; three female and twelve males) and without USN (USN-; n = 15; mean age = 58.5 ± 13.2 years; two female and thirteen males), compared to healthy controls (HC; n = 15; mean age = 61.0 ± 11.3 years; eight females, seven males)	A 3D virtual viewer-centered environment presenting a 9 × 15 mt room displaying walls and ceiling, created using Softimage XSI®. And controlled by the real-time CAREN-3™ (Computer Assisted Rehabilitation Environment). A helmet-mounted display (HMD—NVisor™) displayed the scene, with a binocular field of view of 60° diagonal, 30° vertical by 40° horizontal, Extended Graphics Array resolution. The dominant (non-paretic) hand moved a joystick which controlled a pointer representing the individual, first-person view	The navigation scene included three conditions: (i) online (navigation toward a target always present); (ii) offline (navigation toward a remembered target, present at first, then disappearing); (iii) online (navigation toward a shifting target). The tasks were performed while sitting in order to avoid confounding gait-related effects	Post-stroke USN impairs the ability to detect and adapt to a shifting target and therefore spatial navigation, even in a seated position. Patients displayed a left-sided navigation deviation, possibly due to the absence of a walking demand. The joystick-driven task could better detect perceptual-motor post-stroke USN abilities while failing to estimate USN impact on actual locomotion. The detection task showed USN-related deficits in contralesional targets’ detection time
Ogourtsova et al., 2018b	Twenty-seven patients divided into two groups: with USN (USN + ; n = 12; mean age = 60.7 ± 9.09 years; three female and nine males) and without USN (USN-; n = 15; mean age = 58.5 ± 13.2 years; two female and thirteen males), compared to healthy controls (HC; n = 9; mean age = 56.3 ± 11.2 years; five females, four males)	2 virtual d scenes (complex and simple) created in the Unity game engines, viewed by means of an HMD, field of view-centered of 60° diagonal, 30° vertical by 40° horizontal, extended graphics array resolution 1024 × 1280, frequency of 60 Hz. A stationary joystick (Attack3) was used to provide responses	Practice trials preceded the actual procedure. Within a VR environment (grocery shopping isle), patients performed: (i) detection task: participants pressed the joystick button as soon as detecting the target or refrain from clicking while absent; auditory feedback followed. A simple and complex scene was presented (30 responses), with one or multiple items placed on shelves. (ii) navigation task: participants had to navigate toward the target in the most straightforward way possible. A total of 25 responses was required for the complex and simple conditions	Compared to USN- and HC, USN + patients showed significantly longer detection times in the detection task and moved toward the target following a “searching strategy” in the navigation task (3-way significant interaction of group x scene complexity x target location). The navigation task outcome (time to target) and the detection time task (detection time) worsened as a function of USN increased severity. Findings show lateralized and non-lateralized deficits in USN individuals
Knobel et al., 2020	A group of 15 patients (mean age = 67.1 ± 17 years) divided into two categories (Neglect versus No-Neglect group); healthy controls (n = 35, mean age = 69.0 ± 15 years)	HMD displaying 20 stimuli (white spheres as targets and white cubes as distractors) disposed within a hemisphere on a blue background. A hand-held controller was used to provide the answers. A mobile Gaming-Laptop was needed to run the software programmed with Unity3D	Patients were divided into two sub-groups after completing the Sensitive Neglect Test. Then, they were asked to find all targets avoiding distractors, randomly displaced in the virtual environment, as fast as possible. Immediate feedback was provided with any object turning red when touched. Finally, they completed a questionnaire on usability and cybersickness	The HMD-based assessment appears slightly less sensitive in detecting neglect symptoms and their severity, compared to the paper-and-pencil task. This could be due to a low number of stimuli and distractors, as well as a reduced sample size. The VR procedure, however, showed very high usability and acceptance among patients
Spreij et al., 2020	A group of 100 patients divided into five sub-groups: 33 patients with left-sided VSN + (n = 33, mean age = 58.83 ± 9.18), right-sided VSN + (n = 7, mean age = 54.75 ± 11.48), left-sided R-VSN (n = 7, mean age = 54.47 ± 14.69), no VSN (n = 53, mean age = 58.86 ± 12.14), and healthy controls (n = 21, mean age 58.77 ± 9.86)	A digitalized version of the CBS. A screen-projected driving task projected on a screen and a steering wheel that patients used during the task	Patients adjusted and maintained their position at the center of the right lane using the steering wheel, despite a simulated side wind coming from both directions. The projection of the driving scene vibrated when patients drove off into the left or the right verge	Left-sided R-VSN deviated more leftward, compared to VSN- patients, but their average position on the road did not differ significantly from the position of left-sided VSN + patients. A moderate positive relationship was found between the average position and VSN severity as measured by the shape cancellation task, whereas a stronger relationship was found with CBS. However, the driving task cannot be used as a stand-alone tool for the assessment of VSN yet
Yasuda et al., 2020	An 80-year-old man with unilateral spatial neglect (USN) combined with motor paralysis, agnosia, and disturbance of attention	Unity Technology software is used to develop a VR space, displayed in a head-mounted device. A PC recorded patient’s response during the task (i.e., the position of the recognized object using coordinate data)	The patient was instructed to respond when he recognized a red sphere within the VR space. The target object randomly appeared in a different location considering (i) the distance from the patient (0.5, 1.0, 2.0, 3.0, 4.0, 5.0, and 6.0 m); (ii) the angle based on the patient’s line of sight (between 36° and 144°); and (iii) three different stages of height (angle of ± 4° with the eye as the basis)	An immersive VR environment allows the creation of a system to evaluate USN that enables the recording and visualization of spatial neglect in both near and far spaces. Performances in the immersive VR system showed the angle of recognition was significantly larger for near space than far space, and the neglected space decreased with height
Siddique et al., 2021	A single group of 14 USN patients aged between 35 and 65 years	Visual Attentional Lite app, installed on mobiles	The app works in: (i) test mode, to detect patients scanning abilities with increasingly difficult tasks. The tasks (e.g., playing fields, clock) require touching as many targets as possible over a short period of time; (ii) practice mode, to help patients moving their eyes, from top to bottom and from left to right, when they touch the targets	The Visual Attentional Lite app appears a promising tool for USN assessment in acute patients
Kim et al., 2021	Stroke patients with a right-brain lesion and HSN (HSN + SS; n = 19, mean age = 54.32 ± 7.40); stroke patients with a right-brain lesion but no HSN (HSN-SS; n = 22, mean age = 49.23 ± 9.99); a healthy control group (n = 22, mean age = 45.41 ± 17.82)	A stereo HMD system (Oculus Rift DK1); a three-dimensional development platform (Vizard 4.0); a desktop workstation running Windows 7 equipped with NVIDIA GTX 760Ti graphics card	The assessment consisted of two conditions: (i) the FOP condition, where patients fixated at a white cross between each trial. (ii) the FOR condition, where a red cross displayed on the screen identified the center of the HMD	The authors considered success rate (FOPR-SR, i.e., the percentage of correct answers) and response time (FOPR-RT, i.e., the interval between target appearance and mouse click). Both SR and RT provide more sensitive quantification of visuospatial function and discriminating FOP and FOR could allow detecting milder forms of HSN. The FOPR test seems a valid tool for the assessment of visuospatial function but should be integrated with an eye-tracking system

#### Non-immersive technologies

Among the studies included, two papers exploited (Pallavicini et al., 2015a; Siddique et al., 2021) the potential of a mobile device, such as a tablet or an iPad, which at the same time has the technological requirements for supporting VR environments, is affordable and user-friendly. Moreover, the iPad can display traditional cancellation tests and allow to present patients with a digitalized assessment. Despite the paucity of studies considering this tool, Pallavicini et al. (2015a) have developed Neglect App, an iPad-based assessment tool for screening USN symptoms (see also: Cipresso et al., 2018b). The study aimed at exploring the potentiality of the application, consisting of two categories of tasks: (i) Neglect App cancellation tests, which provides a digitalized version of paper-and-pencil cancellation tests and include: *simple cancellation tests*, corresponding to the line cancellation test; *cancellation with distractors tests,* based on the star cancellation test. (ii) Neglect App card dealing task, which recreates a digitalized version of the card dealing task (Pallavicini et al., 2015a). Both correct answers and omissions were recorded. Following a clinical interview and a neuropsychological assessment, the 16 patients selected were assigned to either a neglect group (patients with USN) or a non-neglect group (patients without USN). The assessment procedure consisted of a single session lasting one hour, where patients completed both the paper-and-pencil tests and the Neglect App tasks, in a randomized order. Each session began by administering a self-report scale to evaluate the individual’s technological skills and each subject completed a 10-min session in a virtual environment to get acquainted with the technology, before starting the Neglect App. At the end of the session, each patient filled a System Usability Scale (SUS; Brooke, 1996) to evaluate the usability of Neglect App. Preliminary evidence showed the feasibility of Neglect App for assessing USN symptoms, with the Neglect App version of the cancellation tests proving equally effective to the paper-and-pencil version. Specifically, the Neglect App Card Dealing task proved more sensitive in the detection of neglect symptoms, compared to the traditional neuropsychological task. With respect to the cancellation tests, USN patients reported a significantly more aberrant search performance compared to the non-USN group in the virtual cancellation task. With respect to the Card Dealing Task, instead, the USN group reported a significant difference in the omission rates, which decreased only in the Neglect App version of the Card Dealing Task, but not in the paper-and-pencil version, compared to the non-USN group reporting higher omission rates. This latter result could be due to a specific App feature; moreover, the Neglect App version of the Card Dealing Task could assess the near extra-personal space to a higher extent than the paper-and-pencil version.

Jee and colleagues evaluated the feasibility of a semi-computerized version of the classical line bisection test (LBT), for improving the quality of unilateral visual neglect and excluding possible confounding errors deriving from a manual correction of the paper-and-pencil test (Jee et al., 2015). The e-system included an electronic pen (e-pen) capable of recognizing position patterns, a micro-pattern printed paper and a line bisection test software, installed on a computer. The e-pen can detect and send written/drawn information from the micro-pattern printed paper to the LBT software installed on the computer, and it consists of a pressure sensor, an infrared LED, image sensor, a digital signal processor and a Bluetooth interface (Jee et al., 2015). The e-system was previously tested on a group of healthy participants and further applied to a sample of eleven patients with unilateral visual neglect. Specifically, patients were asked to use their right hand to cut each line in half, drawing a single pen notch without skipping any of the stimuli. The printed paper was taped to the table in front of the patient, to avoid excessive movement. The e-system automatically recorded the time needed to complete the task as well. All patients also completed a neuropsychological battery (the Korean version of MMSE, K-MMSE). Results of the inter- and intra-rater reliability of the e-system showed very high correlations, particularly for the group of patients; results were simultaneously and reliably computed, and the e-system could provide information such as pressure and pen tilting. In this group, the authors also noted different results in the neglected lines between the e-system and the raters, and the e-system was recalibrated in order to increase its sensitivity and rule out system errors. They also outline the need for further studies including a wider range of cognitively impaired patients and greater sample sizes. The semi-computerized LBT assessment seems a promising tool to be integrated in a standard neuropsychological assessment of USN.

A digitalized version of the traditional Posner visual cuing paradigm was replicated within a VR context by Guilbert et al. (2016). For the purpose of this review, this paradigm has been considered as it allows to understand the mechanisms underlying visual orienting attention, seemingly impaired in USN patients. A thorough description of the paradigm is beyond the scope of the present paper and should be deepened elsewhere (Posner and Cohen, 1984; Posner, 1980). This effect reflects exogenous orientation of attention and patients with USN manifest deficits in disengaging, moving and engaging their attention, with endogenous orientation being less compromised, and an early orientation toward the ipsilateral side of the lesion (Bartolomeo and Chokron, 2001; Guilbert et al., 2016). This paradigm has been previously applied on healthy subjects (Spence and Drive, 1994) and Guilbert and colleagues’ adaptation aimed at studying the exogenous orientation of auditory attention, less studied in USN patients (Guilbert et al., 2016). Specifically, the authors explored whether detection and lateralization tasks could identify the mechanisms underlying exogenous orientation of auditory attention, as well as their impairments in USN patients. A plausible hypothesis was that hearing and visual attentional orienting shared an underlying deficit unobservable in patients without USN. The authors considered a total sample of 14 patients, four of which were without USN. The total group was then divided into two sub-groups depending on the task they completed, either the detection or the lateralization task. The experimental setup included a 3D virtual environment presenting the auditory stimuli using headphones. Patients were blinded and provided their responses by using a mouse placed in front of them. At the beginning of each trial, a white noise signaled the beginning and the end of each trial (4020 ms) followed by a cue (a pure sound of 1000 Hz, for 20 ms). The target, instead, was a complex harmonic sound of 500 Hz, lasting for 100 ms. The detection task required patients to press a mouse button as soon as they heard the target, whereas in the lateralization task patients had to press either the left or right mouse button following the spatial position of the target. Catch trials (cues in absence of targets) were also present. Results of the detection task found no evidence that either patients, with or without USN, and healthy controls recruited a spatial process to detect a sound; furthermore, no evidence was found for significant cueing effects. Previous studies hypothesized that USN does not imply an impaired auditory detection, because the bilateral projection of auditory stimuli in the brain does not rely on spatial location. Concerning the lateralization task, instead, spatial cuing appeared to have been considered when the task required a spatial judgment to locate the target. USN patients appear to manifest difficulties in auditory orienting. Only one patient performed poorly in this task but was also affected with more severe impairments in sustained attention, which authors did not consider carefully and suggest jointly examining multiple aspects of attention alongside the auditory (Guilbert et al., 2016). However, considering a lateralization task could involve difficulties related to spatial impairment in either processing the response or orienting the auditory attention. Further studies should address the auditory attention orientation, possibly employing the VR-based paradigm suggested by the authors.

The paper by Grattan and Woodbury (2017), instead, compared traditional paper-and-pencil, functional and VR-based neglect assessments to test whether different assessment tools detect neglect differently. Specifically, the VR-based neglect assessment considered was the Enhanced Array Virtual Reality Lateralized Attention Test (VRLAT; Dawson et al., 2008; Buxbaum et al., 2012), a brief assessment tool predicting patients’ collision while navigating in real world. It occurs within a VR environment displayed on a PC laptop, and patients are requested to travel down a virtual path by means of a joystick, while correctly identifying targets (e.g., trees, statues), placed on both left and right sides of the path. Specifically, this version of VRLAT is more challenging due to the multiple distractors (e.g., a ball bouncing in front of the patient, a streetlamp) that patients are instructed to ignore. All patients, suffering from neglect due to ischemic or hemorrhagic stroke, completed the measures in a standard order. They were administered in one or two sessions, either in a laboratory (for outpatients) or in the hospital stroke rehabilitation unit (for inpatients). A complete neuropsychological assessment battery was administered as well (Grattan and Woodbury, 2017). Results showed a greater capacity of both functional and VR-based assessment procedures in detecting patients with neglect, therefore highlighting the different performances on different neglect assessment tools. However, results should be cautiously interpreted due to the reduced sample size (Grattan and Woodbury, 2017).

Spreij et al., (2020) employed a simulated driving task to compare the performance of patients with right- versus left-sided VSN, patients without VSN (“recovered”), and healthy controls. Moreover, they wanted to investigate the relationship between VSN severity and patients’ average position on the road, considered as a measure of lateralized attention. The authors also assessed the diagnostic accuracy of the simulated driving test, compared to traditional tasks. Patients were initially screened by means of neuropsychological measures (CBS, shape cancellation task) and categorized as follows: (i) left-sided VSN + ; (ii) right-sided VSN + ; (iii) left-sided R-VSN (i.e., patients who showed right-sided VSN during the screening but not on the second measurement, considered as “recovered”). Due to the small sample size, the right-sided R-VSN was excluded; (iv) a healthy control group. The setup for the simulated driving task consisted of a driving scene on a straight road, projected on a large screen. A steering wheel was fixed on a table, where a white plain board was placed in order to remove any visuospatial references. After a brief practice trial, patients were asked to use the steering wheel to adjust and maintain their position at the center of the right lane, despite a simulated side wind coming from both directions. The projection of the driving scene vibrated when patients drove off into the left or the right verge. The analysis of group performances showed that, in terms of average position, left-sided VSN + patients deviated more compared to right-sided VSN + and VSN-. Neither right-sided VSN + and VSN- patients nor VSN- patients and healthy controls differed significantly in terms of their average position. Compared to right-sided VSN + patients and VSN- patients, left-sided VSN + patients showed a larger magnitude of sway. Again, neither right-sided VSN + and VSN- patients nor VSN- patients and healthy controls differed significantly in terms of sway magnitude. Left-sided R-VSN deviated more leftward, compared to VSN- patients, but their average position on the road did not differ significantly from the position of left-sided VSN + patients. The authors also found a moderate positive relationship between the average position and VSN severity as measured by the shape cancellation task, whereas a stronger relationship was found with CBS. The authors hypothesized that dynamic tasks requiring a natural behavior and relating more to daily activities, such as the CBS and the driving task, could increase the ecological validity of the assessment (Spreij et al., 2020). However, the sensitivity and specificity values of the simulated driving task indicate that this procedure cannot be used as a stand-alone tool for the assessment of VSN yet (Spreij et al., 2020).

Siddique et al., (2021) employed Visual Attention Lite, a mobile app installed on a tablet, to assess USN on a group of 14 acute stroke patients. The app has two modes: (i) test mode, designed to detect both time and accuracy of patients’ scanning abilities, with increasingly difficult tasks. The tasks (e.g., playing fields, clock) require patients to touch as many targets as possible over a short period of time; (ii) practice mode, designed to help patients move their eyes, from top to bottom and from left to right, when they touch the targets. Each level of difficulty is completed only when the patient finds all the targets. Patients had to complete ten consecutive days of practice using the Visual Attention Lite practice mode. Before and after the ten days of sessions, patients were assessed by means of the Visual Attention Lite test mode. Results showed that the app seems a promising tool for the assessment and management of USN in acute stroke patients.

#### Fully immersive technologies

As previously mentioned, VR-based USN assessment has employed HMDs as well and showed promising results so far. Over the years, the field of USN assessment has benefited from the contribution of Aravind and Lamontagne (Aravind and Lamontagne, 2014; Aravind et al., 2015; Aravind and Lamontagne 2017, 2018), whose studies have created a consistent line of research on neglected patients’ behavior, particularly obstacle detection and avoidance. Some of these studies have also been included in the previous systematic review by Pedroli et al. (2015) (Aravind and Lamontagne, 2014; Aravind et al., 2015). Specifically, the paper of Aravind et al. (2015) created an obstacle avoidance task within a virtual environment to assess patients’ ability to both detect and avoid moving obstacles, approaching from different directions. The tendency to collide with static and moving objects and manifest navigational impairments is a common behavior in VSN; whether the collisions are mostly due to either postural and locomotor impairments following a stroke, or to attentional-perceptual deficits due to VSN remains unclear (Aravind et al., 2015). The authors included three tasks consisting of either detecting an obstacle, avoiding a joystick-driven obstacle, or avoiding a locomotor obstacle. These latter tasks were performed using a VR setup comprising an HMD and a joystick that patients used to navigate within the virtual environment. A detailed description of the procedure and the main outcomes are reported in Pedroli et al. (2015). The authors showed the specific involvement of VSN in the altered behavior of obstacle avoidance: not only do patients display a precise pattern of collision, but they also show a correlation between the distance at the time of detection and at the onset of the avoiding strategy. Thus, patients showed a reduced distance from the obstacle at its detection and, conversely, an increased amount of time for the detection of contralesional obstacles, even though patients performed the tasks while seated. VSN patients also showed a gradient of performance, which gradually worsened with contralesional obstacles approaching. Either the attentional/perceptual bias, the predisposition to initiate environmental visual scanning, or the rightward shift in egocentric representation, observed in VSN, could account for this delayed detection of obstacles and initiation of response strategies. The authors hypothesize that VSN patients underestimate the distance between them and the obstacle approaching on the contralesional side, which becomes much closer than patients think, resulting in collisions. VSN patients also displayed specific (delayed) avoidance strategies consisting of both changing mediolateral deviating and increasing speed. However, this could be influenced by the seated versus walking execution of the task in the two studies. The VR setup appeared an easy and useful tool for the assessment of attentional-perceptual deficits and collision avoidance behaviors in VSN patients.

The obstacle avoidance behavior was later examined in two studies (Aravind and Lamontagne 2017, 2018) comparing VSN + versus VSN- patients performing tasks within a virtual environment displayed via HMD, reflective markers, and a 12-camera Vicon motion capture system. This VR-based setup was employed in a previous study (Aravind et al., 2015). The first study (Aravind and Lamontagne, 2017) tested whether the simultaneous negotiation of moving obstacles and performance of a cognitive task would provide a dual-task, cognitive-motor interference. The authors also hypothesized that both VSN + and VSN- patients would display this interference, which would increase for VSN + performing more complex tasks. VSN + would also manifest a more compromised avoidance performance with obstacles approaching patients from their neglected contralesional side. Patients underwent two conditions: (i) Cognitive Single Task (CogST), an auditory discrimination pitch task (Auditory Stroop) that patients performed while observing the virtual environment from a seated position. This condition included a simple task (i.e., the word “cat” presented in a high versus low pitch) and a complex task (i.e., the words “high”/ “low” presented in a high versus low pitch), the latter requiring greater attention and inhibition skills. Patients simultaneously observed a virtual simulation of a locomotor task and had to verbally denominate the pitch of the sound. (ii) Locomotor Dual task (LocoDT), involving obstacle avoidance while performing the simple and complex cognitive tasks. Results showed that VSN + patients displayed a greater deterioration in locomotor and cognitive performances while dual tasking. In line with their previous study (Aravind et al., 2015), VSN + patients displayed a greater collision rate with delays in obstacle perception. Once again, the attentional/perceptual bias emerged more prominently in VSN + patients explaining their compromised obstacle avoidance strategies and their enhanced risk of obstacle collisions. Moreover, dual-task walking has a significant detrimental effect on both cognitive and locomotor performances of VSN patients and is associated with greater locomotory costs, possibly due to deficits in executive functions. The dual-task condition also delayed patients’ initiation of an avoiding strategy even more and increased collision rates, thus showing the importance of task complexity for VSN patients.

In the second study (Aravind and Lamontagne, 2018), the authors compared VSN + versus VSN- patients on both their changes in heading and head orientation while avoiding obstacles arriving toward them from different directions and reorienting toward the target. A small control group (HC) was included as well. Authors also hypothesized that VSN patients would prefer to orient both their heading and head toward the ipsilesional side rather than to the contralesional side, where they would display increased error rates while heading toward the target. A VR-based setup like that of a previous study (Aravind et al., 2015; Aravind and Lamontagne, 2017) was employed; however, this procedure required patients to walk toward the target while avoiding obstacles. Patients underwent the same locomotor obstacle task, conducted in the same virtual environment as of previous studies (Aravind et al., 2015; Aravind and Lamontagne, 2017). Patients had to walk toward the target and simultaneously avoid the collision with an approaching obstacle; if unavoidable, the collision was signaled with a flashing sign. Furthermore, patients completed (ii) a perceptual task in a seated position, which requested them to press a joystick button as soon as they detected a moving obstacle. Patients also underwent a complete clinical and neuropsychological assessment (Aravind and Lamontagne, 2018). Results showed that VSN- and HC avoided obstacles with a specific strategy, i.e., either deviating to the same side as the obstacle or to its opposite side, thus minimizing the risk of colliding. VSN + patients showed higher collision rates with contralesional static and dynamic objects, which could be explained by the ipsilesional bias occurring in VSN and by patients’ preference to direct their attention toward the ipsilesional space (Posner et al., 1984; Dvorkin et al., 2012). VSN + patients also displayed an attentional/perceptual bias resulting in more time spent with their head oriented toward the ipsilesional side and their tendency to rightward deviate their path while walking toward either static or moving obstacles. Moreover, VSN + patients showed poorer variability in locomotor responses, executive functions (assessed with TMT-B) and ability to reorient toward the goal, compared to VSN- patients (and HC). Therefore, both studies (Aravind and Lamontagne, 2017, 2018) strengthen the VR-based paradigm employed as a useful assessment tool for collision avoidance behavior in VSN patients and the influence of task complexity on cognitive and locomotor performances.

The work of Sugihara et al. (2016) tested a digitalized version of line cancellation tests as well. As opposed to the work of Pallavicini et al. (2015a), previously reported, the authors employ a fully immersive technology exploiting HMDs and comparing USN patients to subjects affected by visual field defects (VFD), i.e., a disorder visual space recognition where patients necessarily capture information by eye movement in an unimpaired visual field and head rotation toward the impaired visual field. The aim was to assess the eye movements of USN versus VFD patients and shed light on the features of spatial recognition impairment. The line cancellation task sheet was blanked firstly on the left side and then on the right side. At first, four USN patients were compared to four VFD patients, and they all completed a line cancellation test without wearing the HMD. Then, patients wore the HMD and started performing four conditions of the virtual line cancellation task: (i) no reduction in the size of the test sheet image, displayed on an LCD screen within the HMD; (ii) 80% reduction in the test sheet image toward the central part of the LCD; (iii) and (iv) 80% reduction toward the right and left of the LCD screen, respectively. All patients performed the line cancellation task following this order. Patients’ single-eye movement was also recorded, while they performed the task, by means of two miniature CMOS cameras. Results showed that VFD patients performed correctly under any condition of the left and right test sheets; specifically, their eyeball position was higher on the left-hand side under the conditions of no image reduction, center image reduction, and left image reduction. On the right-hand side, instead, the eyeball position was higher under the right image reduction condition. USN patients’ answer rates differed depending on the task presentation and the reduction side; their 100% correct performance in the left paper-and-pencil condition significantly dropped to 40% under the no-reduction condition, to 42% in the center/right image reduction, and to 38% in the left image reduction, while wearing HMD (Sugihara et al., 2016). Their eyeball position deviated rightward under all conditions and was particularly high under the center and right reduction conditions. Therefore, HMD did not influence the visual field conditions for VFD patients, despite USN patients performing poorly while wearing the HMD. Authors explained these results showing that VFD patients’ eyeballs tended to stay either over the left sheet (in the no-reduction, center, and left reduction conditions) and over the right side (only under the right reduction condition); this could be because patients generally saw the assessment sheets on the side of their affected visual field and visual search enhanced eye movement compensation. This could have increased the ratio of the left-hand side, whereas the right-hand side ratio could have been increased because the assessment sheets tended to be presented in an unaffected visual field, under the right image reduction condition. USN patients displayed a rightward deviation in the eye movement, compared to VFD patients. USN patients’ decreased performance under the HMD condition was possibly due to the rightward deviated attention of all right-handed patients. Their own hand, displayed on the HMD monitor, hid lines placed on the right side of the test sheet, while they performed the cancellation test from the right-hand side. The HMD condition provided a specific range of attention while diverting it rightward. The major drop was observed for the answers given under the left reduction condition; placing the left sheet to the left side of the screen (under the left image reduction condition) could have contributed to this decrease.

Ogourtsova and colleagues conducted two studies exploiting the potential of VR-based assessment (Ogourtsova et al., 2018a, 2018b). The first study considered the role of spatial cognition on locomotion and navigational abilities in post-stroke USN patients (USN +), presenting with walking deficits in goal-directed conditions with cognitive and perceptual demands (Ogourtsova et al., 2018a). Goal-directed walking deficits could depend on USN-induced perceptual and attentional deficits or could be mediated by post-stroke sensorimotor alterations as well, affecting gait, balance, and posture. The authors compared USN + to non-USN patients (USN-) and healthy controls (HC). They explored the effects of post-stroke USN on controlling goal-directed walking behavior in a goal-directed navigation task carried out with a joystick, within a VR environment displayed by means of an HMD. The navigation task included three conditions: (i) online, where the participant had to navigate toward a target, always visible; (ii) offline, where the participant had to remember the position of a target that disappeared during the navigation task; (iii) online, where the participant had to navigate toward a shifting target, which changed its location following participant’s displacement. The study had several aims, the primary being to estimate the degree to which post-stroke USN affects goal-directed navigation abilities in both conditions. The navigation task was performed while sitting, in order to minimize potential confounding effects of gait and walking abilities, where USN patients differ from a healthy control. Results are in line with a previous study (Aravind et al., 2015): USN presents with specific deficits in both spatial navigation and object detection and a joystick-driven task could reflect real perceptual-motor abilities in neglect. USN subjects showed a greater endpoint mediolateral error in the offline, memory-guided condition. USN patients seemingly manifest an impaired ability to update representation during navigation, as shown by their impaired ability to detect and adapt to a shifting target. Therefore, post-stroke USN appears to have a detrimental effect on spatial navigation, even in a seated position. Contrarily to previous studies, the authors found a left-sided navigation deviation, which could be explained by the absence of a walking demand in this study. The joystick-driven task appeared more useful for detecting perceptual-motor post-stroke USN abilities while failing to estimate USN impact on actual locomotion. The detection task was also able to highlight USN-related deficits in contralesional targets’ detection time, in line with previous research focusing on the attentional theory of USN (Kinsbourne, 1993; Smania et al., 1998).

Later, Ogourtsova, et al. (2018b) developed a three-dimensional Ecological VR-based Evaluation of Neglect Symptoms (EVENS) and examined its feasibility in a cross-sectional observational study. Specifically, the aim was to investigate the effects of post-stroke USN on both object detection and navigation toward a target, within a virtual grocery shopping isle. The VR task was provided by an HMD, requiring USN patients to perform a detection task and a navigation task within a grocery shopping isle. Patients could navigate and interact within simple and complex VR environments by pressing a joystick button. On the one hand, the *detection* task required patients to press the button when they detected a target (e.g., a blue cereal box), either in a simple scene (e.g., the blue box alone on a shelf) or in a complex scene (e.g., the same blue box among other distractors placed on multiple shelves), receiving auditory feedback afterward. In the absence of the target, patients were asked to wait for the next trial. On the other hand, the *navigation* task required patients to navigate toward the target using the joystick, following the most straightforward pathway possible. The VR-based assessment was preceded by a practice trial for patients to get acquainted with the procedure and neuropsychological measures were collected (Ogourtsova et al., 2018b). A repeated-measures mixed model was employed to examine the effects of scene complexity and targets’ location on both detection time and goal-directed navigation. For the detection task, USN + patients showed significantly longer detection times compared to both USN- and healthy controls. For the navigation task, USN + patients moved toward the target after “searching” for it, making corrections at the end of the trial, as opposed to USN- patients following a selected direction from the beginning to the end of the task. Moreover, it was tested whether the outcomes from both the detection and navigation tasks varied as a function of USN severity: specifically, the navigation task outcome (time to target) and, to a lesser degree, the detection time task (detection time) were progressively more affected as a function of USN increased severity. Therefore, USN negatively affects both perceptual and navigational abilities to targets placed on the neglected side, and the deficits worsen to a greater extent when patients are exposed to a complex scene. These deficits in detection time and time to target could be explained within the conceptual framework of the attentional mechanisms underlying USN.

The work of Ogourtsova et al. (2018b) also paved the way for the study of Yasuda et al., (2020). The authors note that the study is mainly focused on neglect in the extra-personal space and was not able to quantitatively identify patients’ neglected areas in the three-dimensional space. Therefore, the aim of the proof-of-concept study of Yasuda and colleagues was to develop and introduce an HMD-based assessment for both near and far space neglect. The setup included an HMD, a personal computer running Unity software for the VR environment, and a tracking sensor. The virtual environment was tested on a single stroke patient and consisted of a virtual room seen from a first-person perspective, in which a target sphere appeared at several angles of distance from the patient, ranging from 50 cm to 6 m. The system recorded the patient’s area of neglect for each distance, while the sphere appeared in concentric circles over the patient’s head. Depending on the line-of-eyesight of the patient, three stages of height were defined. The patient was instructed to verbally identify the presence of the red sphere when he recognized the target in the virtual room, within a 30-s time limit. Results showed that the patient had a significantly larger angle of recognition for near-space than far space and tends to increase the angle of recognition when height decreases. The immersive VR environment seemingly allows to record and visualize both near and far space in USN; however, further studies are needed to confirm the reliability and validity of the platform, as well as its clinical utility.

Knobel et al., (2020) aimed at testing the feasibility and acceptance of an HMD-based visual search task and its ability to detect neglect. A group of 15 patients was divided into two sub-groups (Neglect and No-Neglect) depending on their performance at the Sensitive Neglect Test (SNT; (Reinhart et al., 2016). The setup consisted of an HMD displaying a blue background with 120 objects (white spheres as targets and cubes as distractors), disposed within a hemisphere. The spheres were placed symmetrically, whereas the cubes were randomly distributed across the virtual environment. Patients held a controller to provide their answers and were asked to touch all the targets as fast as possible, without touching the distractors. After the procedure, patients filled an adapted version of the System Usability Scale (SUS; Brooke, 1996) and a measure regarding cybersickness (Simulator Sickness Questionnaire, SSQ; Kennedy et al., 1993). Results showed that both sub-groups of patients did not differ in terms of acceptance, usability, or adverse effects of VR-based assessment. The authors also included the Center of Cancellation (CoC, Rorden and Karnath, 2010) as a measure of neglect severity, assessed in cancellation tasks. This measure reflects the normalized mean deviation from the center, due to neglect, ranging from − 1 to 1, there positive CoC values indicate a rightward shift, and a negative CoC indicates a leftward shift in the space. The CoC measure was analyzed comparing Neglect, No-Neglect, and control group; compared to the paper-and-pencil version, the VR cancellation task seems less sensitive in detecting neglect symptoms and severity. This result could be explained by several factors, such as the different configurations of the three-dimensional VR stimuli versus the two-dimensional, paper-and-pencil version. The authors also hypothesize that the low number of targets and distractors could reduce the sensitivity of the test; therefore, future applications of this method should increase the number the stimuli, as well as the sample size (Knobel et al., 2020).

In a previous study, Kim and colleagues proposed the FOPR previously proposed the FOPR test, i.e., a VR-based assessment for the detection of binocular visual stimuli (Jang et al., 2016; Kim et al., 2021). The FOPR test assumes that individuals need to move their head and body to fully explore a visual scenario and that the visual search patterns can be differentiated into the field of perception (FOP) and field of regard (FOR), depending on the head and body movement. FOP refers to the size or angle of the visual field a person can see without moving the head or the body, whereas FOR refers to the total range of the visual field a person has when moving the head or the body (Jang et al., 2016; Kim et al., 2021). The paper-and-pencil assessment of HSN cannot consider the head and body movement does not distinguish between FOP and FOR, which could be useful to increase the sensitivity of USN assessment. Moreover, considering FOP and FOR could shed more light on the perceptual and exploratory components of HSN. Choosing an HMD allowed to evaluate both FOP (which is unaffected by body movements or head rotations, and the screen is fixed on the patient’s head) and FOR (which requires the exploration of the space as if in the real world), Built-in sensors also allowed to track head movements. The authors considered a group of stroke patients with a right-brain lesion and HSN (HSN + SS), a group of stroke patients with a right-brain lesion but no HSN (HSN-SS), and a healthy control group. HSN symptoms were firstly assessed using the line bisection test and the star cancellation test of the BIT and CBS. Secondly, the FOPR test was administered by means of the HMD and included two conditions: (i) the FOP condition, where patients were instructed to constantly look at a white fixation cross between each trial. As soon as the white cross disappeared, the target was presented (either blue or red spheres). To collect the FOP measurement, the head tracker was turned off in order to preserve the patient’s view of the screen despite the head movements; (ii) the FOR condition, where a red cross displayed on the screen identified the center of the HMD. Patients were asked to move their heads in order to align the red cross and the white fixation cross, before each trial. The FOR measurement was collected when the head tracker was active, and the view of the screen changed according to the head rotation. In both conditions, patients had to click the left or right button of a computer mouse as soon as they saw a blue or red sphere, respectively. Auditory feedback for right and wrong answers was provided. The authors considered success rate (FOPR-SR, i.e., the percentage of correct answers) and response time (FOPR-RT, i.e., the interval between target appearance and mouse click). Results showed that both SR and RT provide more sensitive quantification of visuospatial function and discriminating FOP and FOR could allow detecting milder forms of HSN. Therefore, the FOPR test seems a valid tool for the assessment of visuospatial function. However, the test should be integrated with an eye-tracking system as well and provide an equal number of FOP and FOR tests, possibly using a more ecological virtual environment in order to address patient’s behavior in functional tasks (Kim et al., 2021).

### VR systems for USN rehabilitation

The field of USN rehabilitation has progressively implemented VR-based devices as well: we found 14 articles, nine considering non-immersive technologies (such as computer screens, shutter glasses, joysticks or computer keyboards: Faria et al., 2016; Fordell et al., 2016; Ekman et al., 2018; Wahlin et al., 2019; Tobler-Ammann, 2017a, 2017b; Glize et al., 2017; De Luca et al., 2019; Cogné et al. 2020) and 5 articles including fully immersive technologies (such as HMDs: Kim et al., 2015; Yasuda et al. 2017, 2018; Choi et al., 2021; Huygelier et al., 2020). Table 2 provides a detailed description of the VR technology employed in the following studies, as well as the characteristics of the sample, the sessions, and the main outcomes.

**Table 2:** Technological advancements in neglect rehabilitation. The table briefly summarizes (a) sample size and description; (b) features of the VR device employed; (c) the conditions and tasks of the study; (d) main outcomes

VR AND TECHNOLOG* FOR NEGLECT REHABILITATION				
References	Characteristics of sample	Characteristic of VR applications	Sessions	Main outcomes
Kim et al., 2015	Fourteen patients with hemispatial neglect (n = 14, mean age = 73.1 ± 5.8; nine males, five females)	An amiraglos SX® (Deocom) head-mounted display (HMD); a liquid crystal display (LCD) Screen. OKS and test management software were made with Visual C + + (version 6.0) and Direct X (version 6.0)	Patients performed the line bisection task under several conditions: (i) screen—OKS: patients observed a stationary horizontal red line presented on the LCD screen; (ii) screen + OKS: the red line is presented with background blue OKS moving leftward; (iii) HMD—OKS; (iv) HMD + OKS. These latter two conditions employed the same paradigm as the former two, within an HMD	OKS projected onto a screen overcorrected the hemispatial neglect and outperformed HMD, whereas the OKS + HMD was more effective in decreasing patients' rightward deviation. Leftward HMD + OKS provided a better correction of patients' rightward deviation toward the midline and seemingly distracted patients to a lesser degree while performing the task
Faria et al., 2016	Eighteen USN patients randomly assigned to the experimental group (n = 9, aged 48 to 71 years) or the control group (n = 9, aged 50 to 65 years)	Reh@City, a VR-based simulation of a city, implemented using the Unity 3D game engine; desktop computer running Windows 7, and an arcade joystick (Topway’s Digiusb Joystick)	The experimental group completes increasingly difficult tasks within familiar places (i.e., a post office, a bank, a pharmacy, a supermarket). The cues were gradually removed and reintroduced as soon as they failed to respond correctly. The control group performed a traditional cognitive rehabilitation	The experimental group improved in terms of global functioning, attention, memory and visuospatial abilities, executive functions, social participation, emotion, and in the physical domain. The control group reported a significant worsening in verbal fluency and an improvement in attention and processing speed
Fordell et al., 2016	Clinical trial comparing pre-post-intervention measures of spatial attention in people with chronic neglect due to an ischemic right-sided infarction. 15 participants (mean age = 72.8 ± 5.7 years; 11 males, 4 females)	VR method RehAtt™. 27″ monitor and 3D Nvidia vision glasses. 3D pen and a haptic force feedback interface “Robotic pen” (Phantom omni haptic device) was used as a pointer by the paretic hand. Software developed with the open-source platform Colloseum 3D	5-weeks baseline followed by a 5-weeks training (3 h weekly, 15 h total) with RehAtt™ setup. Follow-up was collected within a week and after 25 weeks. Patients performed one neglect test battery (VR-DiSTRO™); (i) a mental rotation task; (ii) a visuomotor exploring task; (iii) a visuospatial and scanning task	At follow-up, neglect was significantly improved in tests and in spatial attention in activities of daily living. The effect lasted after a 6-month follow-up. The 5-week training with RehAtt™ improved the spatial attention of patients with chronic neglect, transferring the improvements in activities of daily living as well
Glize et al., 2017	Two groups were considered: right-brain-damaged patients with visual neglect (n = 7; mean age = 65.5 ± 4.1 years; six males, one female); healthy controls (n = 10; mean age = 63.3 ± 5.9 years; six males, four females)	Virtual supermarket VAP-S. Participants had to navigate within the supermarket and shop, as quickly as possible, a list of 8 items, 4 placed on the right side and 4 on the left side. The list was randomized on each session. Each session lasted a maximum of 45 min. The PA procedure exposed patients to a rightward optical shift of 10°, produced by the prismatic lenses, by means of glacier goggles (total visual field = 110°; monocular eye field = 80°). The PA session lasted a maximum of 10–20 min, each patient performing 10 sessions over a 2-week period, with at least 200 pointing movements each session. Patients completed the Subjective Straight-Ahead (SSA), pointing straight-ahead with the right arm while the body was aligned with the sagittal axis	Two days of VR training, followed by two days pre-test, patients could familiarize with the virtual environment. The degree of assistance during the performance decreased gradually. Generalization of PA effects to the visuospatial domain, spatial representation, and topographic memory was tested asking subjects to draw the VAP-S map from memory. During the prism exposure, patients had to point visual targets placed on the left or right side of the body midline	PA reduced the rightward attentional bias in a VR task, is possibly associated with an expansion of its effects to a supra-modal representation of space. PA enhanced both navigation and topographic memory, showing a positive effect on access to semantic information. These improvements persist after a 1-month follow-up
Tobler-Ammann et al., 2017a	VSN patients (n = 7, aged 64–78, two females, five males)	A 21-inch computer screen; a height-adjustable chin rest (Novavision GmbH); a haptic Falcon Novint device (Novint Technologies) to control games, providing sensory feedback; 9 increasingly difficult exergames, simulating real-life tasks (e.g., cooking a recipe, completing a puzzle). For the follow-up measurement, the Eye Tracker Neglect Test (ETNT), Zürich Maxi Mental Status Inventory (ZüMAX), and Neglect Test (NET) were administered	Five 30- to 45-min sessions per week, over a 3-week period. The exergames intensity was individually increased by the supervising therapist and patients selected 3 to 4 exergames to perform in each session, following individual preference and were able to change the exergame selected whether they felt bored or preferred another one. A follow-up was conducted after a 4-week break in order to test exergames’ reversibility	The primary outcome showed that neglect exergames are a safe and feasible complementary intervention for VSN patients, with a lack of adverse events and attrition and a 95% median adherence rate. For the secondary outcome (the limited efficacy testing), patients showed a group trend improvement in both cognitive and spatial exploration skills, measured by ETNT, ZüMAX, and NET
Tobler-Ammann et al., 2017b	Two groups of users testing exergames’ usability: VSN patients (n = 7, mean age = 68.6 ± 8.9; five males, two females) and expert group of therapists (n = 12; mean age = 33.3 ± 5.7; gender unspecified)	A 21-inch computer screen; a height-adjustable chin rest (Novavision GmbH); a haptic Falcon Novint device (Novint Technologies) to control games, providing sensory feedback; 9 increasingly difficult exergames, simulating real-life tasks (e.g., cooking a recipe, completing a puzzle)	Both patients (as end-users) and therapists (as experts) completed the 3-week, 30-min exergames training sessions. The intervention included a total of 15 training sessions. After the training completion, patients and therapists completed a Technology Acceptance Model (TAM)-based questionnaire; patients were individually interviewed, and therapists attended two focus group interviews	Patients generally rated exergames as motivating and interesting, despite their initial positive attitude decreasing over time, and games were perceived as boring, childish, or exhausting, also due to the sitting position. Some patients could not understand the purpose of the exergame and preferred conventional therapy. Therapists generally provided lower rates to the use of exergames and more negative attitudes toward them. Therapists had reservations on the therapeutic potential of exergames and reported no intention to use them in the future
Yasuda et al. 2017	Ten USN patients after an ischemic or hemorrhagic stroke, aged between 45 and 85 years. No control group was used	Head-Mounted Displayed (HMD; Oculus Rift Development Kit 2); a motion-tracking device (Leap Motion) and a personal computer running a Unity 5 software. The VR room consisted of a desk and a virtual screen placed in front of the subject. Patients performed the task with their right hand, using a pen in the near-space condition and a pointer in the far space condition	A 30-min session included attentional assessments and two VR training conditions: far space (patients had to orally identify visual stimuli, flashed for 6 s from the right to the left side of the screen); near-space training (patients had to move a VR hand and touch three objects). Attentional assessments included four tasks of the Behavioral Inattention Test (BIT): line cancellation and bisection, letter and star cancellation tasks. The near and far space neglect was assessed as well: the near-space assessment required patients to perform a task using a pen to respond to stimuli presented on a A4 sheet placed at 40 cm. The far space assessment projected the stimuli on a wall and patients were asked to answer using a laser pointer	The far space evaluation showed that total BIT scores improved further after training; three of the four subscales (line cancellation, star cancellation and letter cancellation) improved significantly following the VR rehabilitation and after the session as well, whereas the scores of the line bisection task showed no statistically significant difference. For the near-space evaluation, instead, neither the differences in total BIT scores nor the four subscales were statistically significant. The VR-based rehabilitation appears promising for the rehabilitation of far space neglect in USN patients, but more evidence and a control group are needed for the near-space condition
Yasuda et al., 2018	One male patient (age = 76 years) with left SN after a right middle cerebral artery infarct	Head-Mounted Displayed (HMD; Oculus Rift Development Kit 2); a motion-tracking device (Leap Motion) and a personal computer. Head and finger movements are tracked by both HMD and tracking devices	One 30-min session, once a day, five times a week, over a 6-week training period. Baseline data were obtained 6 weeks pre-intervention and symptoms were assessed pre- and post-intervention. The far space training (10 min) includes a visual search task in the VR space, orally identifying the flashing objects. The near-space training (10 min) required to touch each object presented in the VR space from right to left, followed by a moving slit	The patient showed a major improvement in omission rates for both the line cancellation task in far and near space. An improvement was found in shifting the midpoint in near and far space. No changes were observed in CBS scores, referred to daily life activities
Ekman et al., 2018	Twelve patients with chronic neglect due to right-sided infarction (mean age = 72.7 ± 6.0 years; four females, eight males)	VR method RehAtt™. 27″ monitor and 3D Nvidia vision glasses. 3D pen and a haptic force feedback interface “Robotic pen” (Phantom omni haptic device) was used as a pointer by the paretic hand. Software developed with the open-source platform Colloseum 3D. fMRI data were collected on a 3-T Discovery MR750 Scanner. Patients responded at the Posner cueing task using a response grip (NordicNeuroLab). The behavioral performance at Posner cueing task was recorded with an in-house program in Python (Python Software Foundation)	Two 30-min sessions with a 15-min break, including 5 min of listening to music before audio-spatial training. Patients performed 3 training sessions each week, 5 weeks total. Patients also underwent fMRI scanning 1 week before and 1 week after the VR training and performed a Posner cueing task during the scanning. Patients were presented with two empty boxes at the left and right sides of a red fixation cross, which turned green when the task changed. Patients then had to follow a central arrow cue shifting attention from left to right side of the visual screen (top-down processing) and, later, press a button whenever they targeted a target flashed either on the right or left side of their visual field (bottom-up processing)	RehAtt® training and the Posner cueing task combine an intensive top-down and bottom-up multisensory stimulation implemented within a VR environment. They also show an increased BOLD signal in cortical regions beyond the ventral (VAN) and dorsal (DAN) attentional networks, such as DLPFC, ACC, and bilateral temporal cortex. RehAtt® training is thus capable of inducing neuronal changes in patients with chronic spatial neglect
De Luca et al., 2019	A 57-year-old woman affected by USN following a subarachnoidal hemorrhage in the right frontal–temporal-parietal region	BTS NIRVANA is a movement-based VR semi-immersive system. The standard BTS NIRVANA equipment includes one or two marker-less infrared sensors, a touch-screen workstation, the camera supports, the BTS NIRVANA software, and Webcam. A projector is also connected to a big screen displaying a series of interacting exercises	BTS NIRVANA required the patient to complete two rehabilitation training: standard cognitive training (SCT) was either combined with a semi-immersive virtual training with the patient’s shadow (S-IVT_s) or without the patient’s shadow (S-IVT). Each training combination (SCT + S-IVT_s and SCT + S-IVT) lasted for one month, with twenty 45-min rehabilitation sessions, fine times a week. The motor performance was also of interest and evaluated with the Trunk Control Test. The SCT + S-IVT_s condition included both ideo-motor and attentional tasks while watching her shadow performing them. In the SCT + S-IVT condition, the patient completed similar tasks without seeing her shadow performing them	Following VR-based rehabilitation with the BTS NIRVANA system, the patient displayed improved motor performance, controlling trunk movements, and better cognitive performances. Integrating the S-IVT treatment to the SCT allowed an improvement in attention, scanning, visual search, and spatial cognition
Wåhlin et al., 2019	Patients with visuospatial neglect (n = 13; mean age = 73 ± 6 years; four females, nine males)	The hardware setup included a PC (EMS Shuttle P4), video and sound, a monitor, 3D vision glasses (Nvidia), a robotic pen (Phantom Omni), and a numeric keyboard. The software employed for RehAtt™ used the open-source platform Colloseum3D VRlab	Patients underwent two fMRI scanning sessions, one week before the intervention and one week after the end of the intervention. Within fMRI, patients performed a scanner-adapted Posner task. Symptoms were assessed three times at baseline and at follow-up after the training. The intervention included a 5-weeks training (3 h weekly, 15 h total) with RehAtt™ setup (Fordell et al., 2016)	The intense scanning VR training increased DAN inter-hemispheric functional connectivity in patients affected by chronic visuospatial neglect: this suggests that chronic conditions as well could benefit from training that showed their positive effects for recovery from acute states of neglect. Moreover, the training increased the integration of the frontal eye field (FEF), controlling the saccadic eye movements to the left side of the space, with the left posterior parietal cortex
Choi et al., 2021	Twenty-four post-stroke patients randomized into two groups: a Digital Practice group (n = 12, mean age = 63.00 ± 10.02); a control group (n = 12, mean age = 61.58 ± 9.99)	The virtual environment was created by means of the Oculus Rift Developer Kit 2, with the Oculus Rift 1.3.2 Software Development Kit (SDK) and Windows Runtime 0.8.0-beta. Patients wore Oculus Rift DK2 and used the Leap Motion controller	The experimental group underwent twelve 30-min sessions of digital practice, whereas the control group underwent twelve 30-min sessions of conventional USN rehabilitation	Preliminary results show that only the experimental group reported greater recovery of cognitive and visual perception, as well as self-awareness of neglect. Digital practice could help patients increase their degree of arousal and attention, rotating their heat to a greater degree, with positive effects of attention and arousal on the contralesional side
Cogné et al. 2020	Forty-eight individuals divided into three groups: with unilateral auditory and visual neglect post-stroke (n = 22; mean age = 65.8 ± 8.8 years; 17 males, 5 females); without visual/auditory neglect post-stroke (n = 14; mean age = 63.9 ± 15.5 years; 9 males, 5 females); healthy controls (n = 12; mean age = 67.6 ± 10.0 years; 7 males, 5 females)	A 3D virtual environment reproducing a medium-sized North American town, displayed on a laptop. Participants were able to navigate the town by means of a joystick and a Tobii Pro TX300 eye tracker that detected eye movement. An earphone provided auditory cues in the navigational task	Randomized exposure to either one of the three conditions: (i) “without auditory cues”; (ii) “with auditory cues”; (iii) “auditory cues after prism adaptation”. The prism adaptation procedure itself involved three steps: (i) pre-exposure baseline measurement of pointing; (ii) exposure to prismatic displacement; (iii) post-exposure after effect measurement. A secondary task required a free recall and recognition of landmarks among a pictorial list including distractors. A tertiary analysis considered eye saccades and eye-fixation duration	Primary outcome: auditory cues had a positive effect on spatial navigation abilities in patients with visual and auditory neglect and were even more helpful after a single prism adaptation exposure. Secondary outcome: auditory cues decreased spatial memory abilities, which were compensated following the prism adaptation procedure. Tertiary outcome: the eye-tracking device showed increased duration of the eye fixation, following prism adaptation procedure
Huygelier et al., 2020	Phase 2 and 3 of the study recruited seven stroke patients aged 44 to 69 years	The Oculus Rift CV1 headset with integrated headphones and infrared sensors. The Oculus Touch Controller was used to provide responses; the VR game was developed in Unity 3D	The game world consisted of three scenes (lake, garden, and forest) presented in three lighting conditions (day, evening, and night), for a total of 9 possible combinations. Half of the trials presented a cue that predicted the location of the target. Then, the target was presented for 3 s, and patients pressed the button that corresponded to the target they saw while receiving visual and auditory feedback	The rehabilitation game is a promising tool for detecting visual neglect and improving patients’ performance in orienting their attention to the neglected side

#### Non-immersive technologies

The study of Faria et al., (2016) tackles a relevant issue: cognitive rehabilitation for USN is currently directed toward specific cognitive functions, such as memory, attention, executive functions and language. However, performing daily activities inevitably requires combining them: therefore, a rehabilitation program should account for this complexity and focus on the patient’s global functioning, with ecological tasks and environments reproducing life-like situations. The authors conducted an RCT with 18 stroke patients, divided into two groups; the experimental group underwent a 12-session VR-based intervention with Reh@City (Faria et al., 2016). Reh@City allows an integrative rehabilitation of multiple cognitive domains, personalized with respect to the patient’s needs. The 20-min sessions were distributed over a period of 4 to 6 weeks and patients are asked to complete increasingly difficult tasks within familiar places, such as a post office, a bank, a pharmacy and a supermarket. The virtual city is displayed by means of a computer screen and a joystick enabled the navigation in the environment; if necessary, patients were able to ask for help and several cues were available (e.g., a mini map of the city, a guidance arrow). Whenever patients performed correctly, some of the cues were gradually removed session after session and reintroduced as soon as they failed to respond correctly (method of Decreasing Assistance, DA; Faria et al., 2016). The control group, instead, performed a traditional cognitive rehabilitation. Both groups underwent a cognitive and functional assessment before and after the intervention. Results suggest that VR-based, ecologically valid cognitive rehabilitation programs could be more effective, compared to traditional training. In line with previous studies, the authors report that patients in the experimental condition improved significantly in terms of global functioning, attention, memory, and visuospatial abilities, as well as executive functions (Kim et al., 2011a; Faria et al., 2016). In terms of cognitive functions, the control group reported a significant worsening in verbal fluency and an improvement in attention and processing speed, but this result might be influenced by the fewer years of education of the controls. The experimental group also reported a significant improvement in terms of social participation, emotion, and in the physical domain, suggesting the benefits of a comprehensive rehabilitation outside mere cognition. Despite the promising potential of this rehabilitation program, future studies on Reh@City should include bigger sample size and develop parallel versions for multiple assessments, to avoid learning effects. It would also be relevant to assess the extent of transferring improvements from VR to daily life (Faria et al., 2016).

Three studies (Fordell et al., 2016; Ekman et al., 2018; Wahlin et al., 2019) examined the effectiveness of an integrated platform that includes VR-DiSTRO™ a test battery for assessing neglect, and RehAtt® as a rehabilitation program (Fordell et al., 2016). Specifically, patients completed five computerized neglect tests that assessed spatial attention (Star cancellation test, Baking tray task, Line bisection, Extinction and Posner task). This first testing procedure was carried out using a computer monitor and shutter glasses providing stereoscopic vision. The VR-DiSTRO™ battery test has been previously validated (Fordell et al., 2011; Fordell, 2017). For the purpose of the present review, however, we will consider the VR-based rehabilitation procedure: patients underwent a 5-week VR-based intervention combining multi-modal sensory stimulation, an intense visual scanning training, and a sensorimotor activation while playing 3D videogames (Fordell et al., 2016). The RehAtt® training was firstly developed and employed on patients with chronic neglect due to right side cerebral infarction presenting impaired attentional networks. The primary aim was to provide a recovery method for attention, by stimulating neglect-related mechanisms within and between the ventral (VAN, includes top-down processes) and dorsal (DAN, includes bottom-up processes) attentional network, involved in reorientation, spatial attention, and stimulus selection (Arrington et al., 2000; Corbetta et al., 2005; Corbetta and Shulman, 2011; He et al., 2007; Knudsen, 2007; Ekman et al., 2018).

In their study, Fordell and colleagues (2016) employed the RehAtt® training on 15 patients after they completed a 5-week baseline. The training included both a top-down scanning training with a bottom-up tactile stimulation and visuomotor training. Patients had to wear 3D vision glasses while facing a 27″ monitor and using a robotic pen with a haptic force feedback interface. When playing the VR intervention game, patients used the paretic left hand to grasp the robotic pen, which provided vibrotactile feedback. Patients employed the robotic pen to move, rotate and manipulate 3D objects to perform three different tasks: (i) a mental rotation task, moving 3D figures from left to right side of the screen, placing them on the correct template figure. By means of the robotic pen, these figures could be touched and rotated; (ii) a visuomotor exploring task, picking 3D cubes from a tray located at the right side of the screen. Patients could place the cubes in three-dimensional straight lines; (iii) visuospatial and scanning task, where shapes targets appeared at increasing speed from either above or the left side of the screen, and patients could use the robotic pen to trap, rotate and place them within a puzzle of corresponding shapes. Each 30-min session was separated by five minutes of audio-spatial training. Results showed that the RehAtt™ rehabilitation method could improve elderly patients’ spatial attention performances, and results were both transferring the improvements in activities of daily living and lasting at a 6-months follow-up.

The following studies provided further evidence of neuronal changes following RehAtt® training, by means of fMRI. Specifically, Ekman et al., (2018) tested whether clinical improvements following RehAtt® training resulted in neural changes in attentional networks and related areas, examined by means of fMRI. The authors used the Posner cuing task and evaluated fMRI blood oxygenation level dependent (BOLD) changes before and after RehAtt® training, expecting training-related neuronal changes either within and/or between the ventral and dorsal networks. The authors also hypothesized that RehAtt® training could lead to changes in prefrontal regions, associated to goal-directed behaviors and guiding attention. Twelve patients with chronic neglect following right-sided infarction underwent the same 5-week RehAtt® training as the one described by Fordell et al. (2016). One week before and one week after the training, patients also underwent an fMRI scanning while performing a Posner cuing task. Results showed an increased BOLD signal following the RehAtt® training during top-down focus of attention, where the strongest effects were observed in prefrontal regions associated with goal-directed behaviors and guiding attention (specifically, dorsolateral prefrontal cortex, anterior cingulate cortex and bilateral temporal cortex). Thus, RehAtt® training induces cerebral changes beyond the mere DAN/VAN nodes an expanded to the frontal eye field and ventral frontal cortex as well. Combining an intensive learning of a top-down scanning strategy with a multisensory bottom-up stimulation, implemented in a virtual environment, appears a promising rehabilitation intervention for patients with chronic spatial neglect. However, it should be noted that the present study did have a small sample size and lacked a control group, thus generalization to other neglect patients should be cautious; moreover, estimation accuracy could have been influenced by movement corrections and including brain lesion voxels.

Following this line of research, Wahlin et al. (2019) considered pre-post-rehabilitation improvements in resting-state inter-hemispheric functional connectivity within the DAN following the RehAtt® training. Moreover, the authors adapted a recent tracking method to detect stroke-related longitudinal changes in functional connectivity within resting-state networks. It was examined whether this adaptation allowed to compare changes in DAN to those in other cerebral networks. Thirteen patients affected with visuospatial neglect underwent two fMRI scanning sessions, one week before the RehAtt® training and one week after the end of the intervention. Furthermore, symptoms’ stability was assessed three times at baseline and at follow-up. DAN localizations within fMRI were obtained by means of a scanner-adapted Posner task. Results showed that the intense scanning VR training enhanced resting-state functional connectivity in patients’ DAN, inducing changes in intrinsic neural communication. The tasks included in the intensive training strongly activated neurons within the lateral prefrontal and superior parietal cortices, increasing the functional connectivity between these regions. The intense scanning VR training increased DAN inter-hemispheric functional connectivity in patients affected by chronic visuospatial neglect; this suggests that chronic conditions as well could benefit from training that showed their positive effects for recovery from acute states of neglect. Moreover, the training increased the integration of the frontal eye field (FEF), controlling the saccadic eye movements to the left side of the space, with the left posterior parietal cortex. The authors also explored whether an increased prefrontal activation was observable following the fMRI Posner task. Whereas this effect was not detected within the DAN in a previous study (Ekman et al., 2018), the present study showed an intrinsic DAN change observable in the resting-state condition, and it is plausible that a simultaneous activation within the DAN would provide a major improvement in its intrinsic communication. However, the authors also point out the lack of a control group and the small sample size, which reduces the possibility to carry out more detailed comparisons between sub-groups of patients (Wahlin et al., 2019).

Two studies considered exergames, i.e., a term combining “exercise” and “game” that indicate any type of video games and/or multimedia interactions that require the player to physically move in order to play, thus representing a form of exercise (Oh and Yang, 2010; Tobler-Ammann et al., 2017a, 2017b). Exergames’ design should consider therapeutic and exercise training principles, such as specificity, progression or shaping, providing feedbacks, and tailoring the intervention on patients’ needs (Oh and Yang, 2010, 2017b; Tobler-Ammann et al. 2017a). Specifically, Tobler-Amman and colleagues tested exergames’ feasibility and usability as a plausible complementary treatment for visuospatial neglect (Tobler-Ammann et al. 2017a, 2017b). The authors based their studies on a former version of VSN exergames (Mainetti et al., 2013; Sedda et al., 2013), which showed a trend for improvement of the VSN-related real-life impairments, as well as a positive attitude toward exergames. Tobler-Ammann and colleagues conducted a first quasi-experimental to test exergames’ feasibility and document their effects on early stroke inpatients and VSN symptoms, in terms of intervention implementation (considering treatment adherence, attrition, and safety) and limited efficacy testing. The study did not include a control group to test the feasibility of the full implementation of the procedure. Seven VSN patients completed five 30- to 45-min exergames sessions per week, over a 3-week period; after a 4-week break, a follow-up measure tested whether the performance decreased after removing the stimulus (i.e., the exergame). Each patient autonomously completed an intervention program consisting of 9 exergames, whose difficulty was gradually increased by the supervising therapist, following patients’ performance. The exergames were designed to simulate daily-life tasks, such as cooking a recipe or completing a puzzle (see Pirovano et al., 2016 for a detailed description of the tasks); they were displayed on a 21-inches computer screen which patients could look at in a seated position while placing their head on a height-adjustable chin rest mounted on the table. Moreover, patients used their unaffected hand to handle a haptic device to reach and grasp virtual objects; this device provided sensory feedback as well (Baud-Bovy et al., 2015). The collaborating staff was asked to document the training and completed a diary, specifying treatment adherence, attrition, possible adverse events, and safety issues, while the participant was present. A secondary outcome was to study limited efficacy testing and documenting possible effects on VSN symptoms; authors employed the Eye Tracker Neglect Test (ETNT; Rabuffetti et al., 2012), the Zürich Maxi Mental Status Inventory (ZüMAX; Tobler-Ammann et al., 2016), and the Neglect Test (NET; Halligan et al., 1991), administered at follow-up. Concerning the primary outcome, results showed no dropouts nor attrition and adverse events during the training, and patients showed a 95% median adherence. The exergame intervention appeared well-tolerated by patients and performed without little need for assistance, thus showing its feasibility for clinical implementation. Specifically, the exergame training duration was only 30-min long, possibly due to patients’ fit or fatigue; authors outline that the optimum duration and patterning of training exposure to VR-based environments are not clearly defined and future studies should address this issue. The lack of adverse events was possibly due to games’ design and the seated position from which patients performed them, preventing falls. The secondary outcome, the limited efficacy testing, showed a group trend improvement in both cognitive and spatial exploration skills, which could be partially explained by the ongoing VSN treatment while patients were in the clinic and by the spontaneous recovery from VSN symptoms during the following weeks. Therefore, the neglect exergames intervention proved safe and feasible for VSN patients, most of which improved their cognitive and spatial exploration skills post-treatment, as measured by ETNT, ZüMAX, and NET. It is plausible to evaluate exergames’ implementation in a home-based setting, as a complementary intervention for VSN patients. The authors later tested exergames’ usability with two groups of users; seven VSN patients (affected by an ischemic or hemorrhagic right-sided brain lesion, RBL) and 12 therapists (Tobler-Ammann et al., 2017b). Patients simultaneously participated in a feasibility study testing exergames and in the usability study. The same exergame platform and devices as in the previous study were used and participants could test the exergames before enrolling. At the end of the exergame intervention, both patients and therapists completed a questionnaire based on the Technology Acceptance Model (TAM), which comprises three subcategories: “perceived-user friendliness”, “attitude toward using the exergames” and “intention to use exergames in the future” (Masrom, 2007). Moreover, patients were individually interviewed, and therapists attended two focus group interviews. Patients and therapists agreed on positively judging the “perceived-user friendliness” subcategory of the TAM questionnaire. Despite considering the manual quite understandable and feeling capable of presenting the exergames to the patients, therapists’ attitude toward exergames was negatively influenced and they outlined that independent use of the exergame platform was possible only for some patients, whereas others had difficulties in understanding exergames’ purposes. This latter difficulty led most patients to prefer conventional therapy, which was perceived as more effective. Moreover, despite finding exergames motivating and interesting, patients progressively felt the games were boring or childish and lost interest. The chin-rest support was perceived as exhausting or problematic by both patients and therapists, which had reservations on the therapeutic potential of exergames. Furthermore, neither patients nor therapists reported any intention to regularly use the exergames in the future (Tobler-Ammann, et al., 2017a, 2017b).

The study of Glize et al. (2017) tested whether prism adaptation (PA) could decrease the rightward attentional bias in a VR task, while improving neglected patients’ topographic and navigational memory. Patients’ visual neglect was assessed by means of five paper-and-pencil tests at baseline, before and after each PA exposure, to assess the improvement of neglect symptoms. The VR task was completed within the virtual supermarket VAP-S (Klinger et al., 2006), projected onto a screen placed in a dark room. Patients could navigate within the supermarket with a standard computer keyboard and were asked to enter the supermarket, find items on a shopping list (randomized at each session), place them in the shopping cart and purchase them, as quickly as possible. Before the first pretest, patients could get acquainted with the VR scenario and were gradually less assisted. Each session measured the total distance and duration of the session, along with the number of omissions (i.e., the patient neglecting an item of the list), the total number of items purchased and those purchased on the left or right side, and the number of pauses. Furthermore, the generalization of PA effects to the visuospatial domain, spatial representation, and topographic memory was tested asking patients to draw from memory the supermarket map, without giving any feedback. The PA procedure exposed patients to a rightward optical shift of 10°, produced by the prismatic lenses, by means of glacier goggles enabling a wider binocular vision. Patients were asked to point, rapidly but comfortably, at visual targets presented 10° to the left or the right of the body midline. The PA session lasted a maximum of 10–20 min, each patient performing 10 sessions over a 2-week period. Finally, patients performed a simple manual pointing test, the subjective straight-ahead (SSA), in order to evaluate proprioceptive adaptation. Results indicated that PA reduced the rightward attentional bias of neglected patients, possibly explained by the leftward reorientation of patients’ attention by means of prisms. The reduced rightward attentional bias could be manifested with the expansion of PA effects to higher-level spatial representation and visuo-constructive skills. Despite the direct effect of PA on navigational skills being unclear, PA seemingly improved attentional resources and exerted a positive effect on topographic and semantic information, as if its beneficial effects in processes of spatial localization could extend to the recall of semantic knowledge as well. These improvements persisted after a 1-month follow-up (Glize et al., 2017).

A single-case study considered a post-stroke USN patient, undergoing an intensive cycle of rehabilitation combining standard cognitive training (SCT) and a **s**emi-immersive virtual training (S-IVT) employing the BTS NIRVANA system (De Luca et al., 2019). The patient underwent two rehabilitative pieces of training, the first including SCT + S-IVT with her shadow (S-IVT_s), and the second consisting of an SCT + S-IVT without her shadow. Using the patient’s shadow (projected on a screen placed in front of her) allowed to create her avatar; in the second condition, the lighting conditions were modified so the shadow was not visible, and the virtual environment was more immersive. In the S-IVT_s condition, the patient had to perform increasingly more complex ideo-motor sequences, following the therapist’s instructions. Moreover, the attentional processes have been tackled with increasingly difficult exercises, requiring the patient to select elements observed in the virtual environment. The patient had a limited amount of time to touch the visual target and either a positive or a negative reinforcement was provided. In both cases, the patient observed her shadow while performing the tasks. Furthermore, the authors evaluated whether S-IVT training had an impact on the P300 wave, a positive event-related potential (ERP) component which modulates attention in post-stroke USN (Becker and Shapiro, 1980). Results showed that following VR-based rehabilitation, the patient displayed an improved motor performance, controlling trunk movements, and better cognitive performances. Integrating the S-IVT treatment to the SCT allowed an improvement in attention, scanning, visual search, and spatial cognition. Furthermore, results showed an increased amplitude in P300 correlated to improved cognitive functioning following the S-IVT training (De Luca et al., 2019). However, the persisting effect of this intervention should be further examined with greater sample size.

Another digital adaptation of a traditional rehabilitation method, such as prism adaptation (PA) has been tested in an exploratory, prospective randomized controlled study by Cogné et al. (2020). A thorough description of the PA paradigm, previously mentioned, is beyond the scope of this paper and should be deepened elsewhere (Jacquin-Courtois et al., 2010). The study conducted by Cogné and colleagues involved patients suffering from post-stroke visual and auditory neglect and evaluated whether lateralized cueing, before and after a PA procedure, improved virtual spatial navigation (Cogné et al., 2020). As for secondary and tertiary aims, the authors evaluated the possible effects of lateralized cueing in improving patients’ spatial memory and gathered further information on auditory cueing treatment, using an eye-tracking device, respectively. Patients were randomly exposed to either one of the following conditions: (i) without auditory cues; (ii) with auditory cues; (iii) auditory cues after prism adaptation (Cogné et al., 2020). Patients were exposed to a 3D virtual environment displayed on a laptop, reproducing a North American town, within which they could navigate using a joystick. A familiarization task preceded the actual procedure, which consisted of three 15-min evaluation sessions; initially, participants passively watched a path that, in the reproduction phase, had to actively retrace. Subsequently, participants were asked to observe and then replicate two different paths, whose order was randomized, with auditory cues providing information on the direction to take at each intersection (left, right, straight-ahead). The third condition required patients to trace a path following auditory cues after completing a PA session. The latter consists of a 10-min, three-step procedure, specifically pre-exposure baseline measurement of pointing; exposure to prismatic displacement; post-exposure after effect measurement. A detailed description of the procedure is provided by Jacquin-Courtois et al. (2010). The secondary outcome was addressed asking participants a free recall and recognition of landmarks, seen throughout the path, among a pictorial list including distractors. Finally, the tertiary outcome was addressed by analyzing eye saccades and eye-fixation duration. Results showed the positive effect of auditory cues on spatial navigation abilities in patients with visual and auditory neglect; moreover, auditory cues helped patients in the navigational task even more after a single PA exposure. Patients with neglect appear capable of detecting and treating lateralized auditory cues in order to navigate more efficiently. Thus, auditory cues could provide directions and guide neglected patients in real life as well. Moreover, the present study strengthens the evidence in favor of PA and its ability to improve navigational performances in real-life environments, despite patients performing at a lower level compared to healthy controls. Further effects following a PA exposure were the compensation of decreased spatial memory abilities and improved duration of eye fixation detected with an eye tracker.

#### Fully immersive technologies

Five studies employed HMDs as a rehabilitative tool for USN. As previously mentioned, OKS has proved as an effective rehabilitative intervention for left hemispatial neglect, training patients to either look at or pay attention to stimuli placed in their left (neglected) hemispace (Pizzamiglio et al., 1990; Robertson et al., 1998; Moon et al., 2006; Kim et al., 2015). However, its utility has been limited to experimental settings within a laboratory so far: in order to be effective within a real-life environment, OKS should be projected in the background, which is often impossible or impractical (Kim et al., 2015). VR technology could be particularly useful for this purpose, allowing to administer OKS by means of HMDs, as tested by the preliminary study of Kim et al. (2015) to explore the feasibility of HMD-based administration of OKS. Specifically, the aim was to test whether OKS, displayed by HMD, would be as effective as using background OKS and if so, HMD-based OKS could be useful for treating hemispatial neglect. The added value of HMD lies in its ability to overlap OKS with real-life objects, thus, presenting them simultaneously, which cannot occur with a traditional (screen) display of OKS. Patients were asked to perform a line bisection task under four different conditions: (i) screen—OKS: the LCD screen presented patients with a stationary, horizontal red line, but no OKS; (ii) screen + OKS: the LCD screen presented patients with the same red line, in addition to OKS displayed as blue vertical stripes, moving leftward. The same conditions were displayed on the HMD as well in conditions (iii) HMD – OKS and (iv) HMD + OKS (Kim et al., 2015). Results confirmed previous evidence supporting the beneficial effect of OKS on hemispatial neglect and expanded their applicability to HMD devices. Specifically, these devices could simultaneously overlap OKS and real-life objects, reproduced in a virtual environment, to reduce patients’ distractibility. Results from the screen condition confirmed previous results, showing OKS’ positive effect on hemispatial neglect; however, the study also showed that see-through HMD could be employed as a rehabilitative tool for hemispatial neglect. OKS projected onto a screen overcorrected the hemispatial neglect and outperformed HMD, whereas the OKS + HMD was more effective in decreasing patients’ rightward deviation. The added value of see-through HMD lies in its ability to overlap real objects and projected OKS, an advancement not possible using a screen. OKS could be repeatedly applied to neglected patients’ peripheral visual field to help them see the objects in their central visual field more clearly, and eventually contribute to neural reorganization and reduction in neglect symptoms. Comparing the leftward HMD + OKS vs screen + OKS, the former provided a better correction of patients’ rightward deviation toward the midline and seemingly distracted patients to a lesser degree while performing the task. The screen presentation is wider and could allow a greater distraction due to background movement, however, the authors claim that this effect should be further explored. Overall, the results suggest that the HMD + OKS combination seems promising for the rehabilitation of hemispatial neglect.

Yasuda et al. (2017) conducted a pilot study to tackle the issue of near and far space neglect observable in USN patients, which has not been sufficiently considered by previous research employing VR-based rehabilitation (Ogourtsova et al., 2019). Thus, the authors tested an immersive VR rehabilitative program by means of a HMD, providing a first-person perspective for targeting near and far spatial neglect. The study employed a within-group, pre-post comparison of the extent of near and far space neglect following the VR rehabilitation. No control group was used. The far space training condition employed a virtual screen, on which several visual stimuli were consecutively flashed for six seconds, from the right to the left portion of the screen. Patients had to verbally identify each of them. In the near-space training, patients used their own hands to move a virtual hand and touch three objects presented in the virtual space, from right to left. A moving slit was also included in the VR environment, to promote attention disengagement and shifting from right to left and improve neglect by removing the stimuli in the right side of the space, as shown by previous studies (Mark et al., 1988). The near and far space neglect were also assessed with a “traditional” paradigm: the near-space assessment required patients to perform a task using a pen to respond to stimuli presented on an A4 sheet placed at 40 cm. The far space assessment projected the stimuli on a wall and patients were asked to answer using a laser pointer. Attentional impairments were evaluated by means of four tasks of the Behavioral Inattention Test (BIT; Hartman-Maeir and Katz, 1995): line cancellation, star cancellation, line bisection, and letter cancellation tasks. Results showed a significant pre-post difference in the total BIT scores for far space evaluation, and the scores improved further after training; three of the four subscales (line cancellation, star cancellation, and letter cancellation) improved significantly following the VR rehabilitation and after the session as well, whereas the scores of the line bisection task showed no statistically significant difference. For the near-space evaluation, instead, neither the differences in total BIT scores nor the four subscales were statistically significant. Therefore, the pilot study showed that the VR-based rehabilitation had a positive immediate impact on far space but not on the near-space neglect, suggesting that near and far space is represented differently, in line with previous research (Previc, 1998). The authors also suggest that the VR program was ineffective for line bisection, in far space as well, because this task requires to focus the attention on the horizontal plane of a specific object, as opposite to cancellation tasks that require to explore stimuli placed in random order. It is plausible that the tasks required in this study decreased the effect of attention required for the line bisection task. Overall, this study offers a preliminary, promising VR-based intervention for USN rehabilitation, particularly of far space neglect, despite the lack of a control group and the need for more solid evidence on its use.

In 2018, the authors presented a single-case application of the same VR protocol on a left SN patient, which completed a line cancellation and bisection test and the Catherine Bergego Scale (CBS) as well (Yasuda et al. 2018). The patient then enrolled in a 6-week VR training completing a daily 30-min session, five times a week; baseline measures were obtained 6-weeks pre-intervention and a follow-up was carried out 6 weeks after the training completion. Results showed a marked improvement in patient’s neuropsychological measures, replicating previous findings; however, despite the positive effect of VR-based training, CBS scores (assessing patient’s performance of daily activities) did not change over the course of the intervention. A possibility is that the patient was not able to transfer his visual search skills to far space of daily activities or, alternatively, CBS measures are not sensitive enough to detect VR potential effect in ADL and might be influenced by the patient’s age (Yasuda et al., 2018).

Huygelier et al., (2020) built a rehabilitation game based on several intervention principles that could positively affect neglect recovery. The authors used a tailored, HMD-based serious game rehabilitation program, providing peripheral salient and informative cues to stimulate endogenous and exogenous orientation of spatial attention toward the contralesional side. Building on a previous study (Dent and Humphreys, 2011), the authors used informative peripheral cues, audiovisual and looming stimuli to promote patients’ endogenous and exogenous spatial attention orientation toward the neglected side. The setup included an Oculus Rift CV1 with integrated earphones and infrared sensors, to provide full rotational tracking. Patients could interact with the virtual environment by means of an Oculus Touch controller. The game world consisted of three scenes (lake, garden, and forest) presented in three lighting conditions (day, evening, and night), for a total of nine possible combinations. Each combination was presented twice during the rehabilitation, for a total of 18 levels. After a brief explanation of how to use the controllers, patients were introduced to the game narrative and instructed on a specific task they had to perform, as part of the storyline. The size and amplitude of the visual and auditory cues changed at the same frequency and in phase. For each level of the game, two target stimuli (either in 2D or 3D, different for color and shape) were presented; the position of both cues and targets depended on the HMD orientation at the beginning of the trial. The virtual game reproduced two variations of a visual discrimination task and half of the trials presented a cue that predicted the location of the target. Then, the target was presented for 3 s: patients had to report which target they saw, by pressing the corresponding button. They received visual and auditory feedback whether the answer was correct, incorrect, or absent. Patients also underwent a complete neuropsychological assessment and a computerized cancellation task, as well as measures regarding cybersickness and user experience. Results showed that the rehabilitation game can detect visual neglect and improve patients’ performance in orienting their attention to the neglected side. No side effects were reported, and the VR setup was positively rated. The authors also outline the two faces of a highly individualized rehabilitation; while it allows adapting to patient’s needs in order to maximize the benefits, it also requires collecting a large amount of data in order to estimate the effects of changing the single-game features (Huygelier et al., 2020). VR-based serious games appear a promising tool for USN rehabilitation.

A single-blind RCT (Choi et al., 2021) integrated an HMD with a gesture recognition system interface, in order to provide a digital practice for USN and to observe its effects on visual perception, ADLs, and degree of neglect. The experimental group underwent twelve 30-min sessions of digital practice, by means of Oculus Rift DK2 and Leap Motion. The Leap Motion controller is a portable marker-less device able to capture hand and finger position, by means of infrared light and cameras. The control group, instead, underwent twelve 30-min sessions of conventional USN rehabilitation, including reading, writing, and puzzles. The assessment conducted pre- and post-intervention included neuropsychological tests, indexes of functional disability and independent functioning, and a visual perception test, as well as the head tracking sensor data. Despite both groups showing improvements in all measures, only the experimental group reported greater recovery of cognitive and visual perception, as well as self-awareness of neglect (Choi et al., 2021). The authors suggest that digital practice could bolster patients’ participation and interest by providing immediate feedback and thus increasing their degree of arousal and attention. Moreover, the VR-based digital practice allows patients to rotate their heat to a greater degree, with positive effects of attention and arousal on the contralesional side. Future research should consolidate these preliminary results.

## Discussion

Traditional paper-and-pencil tools have represented the benchmark for both USN assessment and rehabilitation, and they are currently serving as a focal point in the field of neuropsychology. However, despite their usefulness, these instruments have been criticized for several reasons: they evaluate patients’ cognitive functions as isolated, with abstract stimuli or tasks that do not address patients’ functional performance in their daily-life activities. Moreover, both assessment and rehabilitation for USN are carried out in a clinical or experimental setting that does not resemble the environment in which patients usually live. Therefore, the traditional paper-and-pencil neuropsychological tools often lack sensitivity in detecting milder deficits and ecological validity. Two major transitions have concurred to increase the ecological validity of assessment and rehabilitation procedures: firstly, the paper-and-pencil tools have been converted to a computerized form and displayed by means of non-immersive devices, such as computer screens and keyboards. Secondly, the recent technological advancements in the field of VR have led to the development of fully immersive devices, such as HMDs, creating a higher feeling of immersion and presence and allowing the user a more realistic interaction with the environment. The present paper shows that over the past six years, USN assessment and rehabilitation seem to have benefited from an increasing number of studies exploiting the potentialities of VR technologies, either non-immersive or fully immersive. It is crucial to remind that these devices integrate rather than substitute the traditional neuropsychological assessment and rehabilitation procedures. The following discussion will provide both methodological and clinical considerations on the studies included in the present paper, including a plausible hypothesis for the mechanisms underlying the effectiveness of VR for rehabilitation.

### Technological advancements in USN assessment

#### Methodological considerations

The search strategy performed, following the guidelines of Pedroli et al. (2015), lead to the selection of fifteen studies of VR-based USN assessment (Pallavicini et al., 2015a; Aravind et al., 2015; Jee et al., 2015; Sugihara et al., 2016; Guilbert et al., 2016; Grattan and Woodbury, 2017; Aravind and Lamontagne, 2017, 2018; Ogourtsova et al., 2018a, 2018b; Yasuda et al., 2020; Knobel et al., 2020; Spreij et al., 2020; Kim et al., 2021; Siddique et al., 2021). From a methodological point of view, almost all the studies employed a control group mainly consisting of patients without USN or affected by other conditions (Pallavicini et al., 2015a; Sugihara et al., 2016; Guilbert et al., 2016; Grattan and Woodbury, 2017; Aravind and Lamontagne, 2017, 2018; Spreij et al., 2020; Knobel et al., 2020; Kim et al., 2021) and, in some cases, healthy controls as well (Jee et al., 2015; Ogourtsova et al., 2018a, 2018b; Spreij et al., 2020; Knobel et al., 2020; Kim et al., 2021). Two studies did not consider a control group (Aravind et al., 2015; Siddique et al., 2021). In line with the paper of Pedroli et al (2015), the present review shows that the studies present some methodological issues, mainly regarding the small sample size and the lack of randomized controlled studies. Moreover, despite the technological advancements in this field, normative data on USN assessment are currently lacking and authors have not reached a consensus regarding which tool has the most sensitivity and specificity in detecting USN symptoms.

Six studies employed a non-immersive setting, consisting of either an application displayed on a tablet (Pallavicini et al., 2015a; Siddique et al., 2021), a semi-computerized version of line bisection task (Jee et al., 2015; Spreij et al., 2020) or a computer screen, a mouse/joystick to navigate the virtual environment or to answer, and auditory stimulation provided by speakers/ear cuffs (Guilbert et al., 2016; Grattan and Woodbury, 2017); nine studies employed a fully immersive setting, consisting of HMDs, reflective markers placed on body parts and miniature cameras to detect ocular movements (Aravind et al., 2015; Sugihara et al., 2016; Aravind and Lamontagne, 2017, 2018; Ogourtsova et al., 2018a, 2018b; Yasuda et al., 2020; Knobel et al., 2020; Kim et al., 2021). As previously mentioned, ecological validity is a crucial feature of VR technologies and is particularly relevant for USN assessment and rehabilitation. Therefore, we have compared the virtual environments employed in the research considered in the previous work of Pedroli et al. (2015) to those included in the present paper. This has allowed us to evaluate whether a VR-based USN assessment could be considered genuinely more ecological than the traditional procedure. The present paper shows that among the non-immersive VR setups considered, the VRLAT appears the most ecological and realistic virtual environment, despite some of the targets that might seem unusual (e.g., the statue of a cow or a pig) (Grattan and Woodbury, 2017). Another non-immersive study employed a simulated driving task, and the authors suggest that dynamic settings that require patients to perform life-like movements in daily situations could enhance USN assessment and provide more ecological validity (Spreij et al., 2020). The authors also suggest that extending the traditional assessment with dynamic tasks would allow detecting VSN in its diverse manifestation (Spreij et al., 2020). The other studies employing a non-immersive setup recreated either a three-dimensional version of traditional neuropsychological assessment procedures (Pallavicini et al., 2015a), or were mainly focused on studying auditory attention, thus a particularly rich virtual environment was not required (Guilbert et al., 2016). This latter study, specifically, placed patients’ heads within a square virtual room but masked their eyes to avoid the interference of visual stimuli.

Among the fully immersive VR setups that exploited HMDs, two studies recreated a digitalized version of the Line Cancellation test using a non-ecological environment (Sugihara et al., 2016; Knobel et al., 2020). Four studies (Aravind et al., 2015; Aravind and Lamontagne, 2017, 2018; Ogourtsova et al. 2018a) studied obstacle avoidance behavior within a virtual room with approaching obstacles (e.g., red cylinders, a red ball). The performance of the obstacle avoidance task becomes increasingly more ecological: while two studies ask patients to navigate toward the obstacle using a joystick (Aravind et al., 2015; Ogourtsova et al., 2018a), the other studies ask patients to walk in the virtual room while tracking the movements (Aravind and Lamontagne, 2017, 2018). Despite representing a richly textured and plausible situation that patients might encounter in real life, the environment could be only little representative of daily context. A plausible, more ecological alternative could display environments such as a crossing or a supermarket, as in the study of Ogourtsova et al. (2018b); the authors presented two ecological viewer-centered scenes that displayed a grocery shopping aisle presenting one or more products (in the simple and complex scene respectively), and patients could interact with the scene using a joystick. Overall, it seems that over the years researchers have continued to use virtual environments that appear sufficiently close to daily situations, with a greater focus on patients’ actual behavior and impairments. However, some of the stimuli employed remain quite abstract, particularly in those studies that investigate the obstacle avoidance behavior or the FOR and FOP components of visual functioning (Aravind et al., 2015; Aravind and Lamontagne, 2017, 2018; Ogourtsova et al. 2018a; Yasuda et al., 2020; Kim et al., 2021). Future research could replicate those findings with more realistic stimuli to enhance the ecological validity of the VR-based assessment.

In terms of VR employment for USN assessment, some research included in this section has tried to digitalize traditional neuropsychological tests, and to compare VR-based versus standard procedures to verify its higher sensitivity in detecting USN deficits. This is the case of Neglect App (Pallavicini et al., 2015a), which recreated paper-and-pencil tests (specifically, cancellation tests and card dealing tasks) and showed that USN patients displayed a more aberrant search performance in cancellation tests but in the card dealing test patients showed a difference in decreased omission rates only in the virtual task, possibly due to the digitalized version. A semi-computerized version of the LBT was proposed by Jee et al. (2015), by means of an e-pen and a micro-patterned paper that recorded and sent written information to an LBT software. Despite the innovative method, the e-system could be expensive and should be tested on a wider range of patients to provide more solid results. Furthermore, the traditional Posner paradigm was adapted to a virtual setting to explore the exogenous orientation of auditory attention, possibly compromised in USN patients (Guilbert et al., 2016). This deficit could manifest whenever patients need to perform a spatial judgment to locate a target, whereas their auditory detection skills seem intact. However, more studies are needed within this field and the VR-based environment could be particularly useful for this purpose. Finally, one study globally considered how different assessment tools could detect neglect differently (Grattan and Woodbury, 2017) and compared paper-and-pencil, functional, and VR-based neglect assessments. Both functional and VR-based assessments appeared the most capable of detecting patients with neglect, despite further research is needed to consolidate this finding, due to the small sample size. Only one study evaluated a novel HMD system for a quantitative assessment of visual recognition abilities and eye movements of USN versus VFD patients (Sugihara et al., 2016). Despite VFD patients performing correctly under any condition (either paper-and-pencil or HMD), USN patient’s performance dropped significantly when performing the HMD-based assessment, compared to the paper-and-pencil version. Moreover, USN patients displayed a rightward deviation in eye movement, compared to VFD patients. The new HMD-based assessment system developed could be a promising tool to objectively assess the disturbance in visual space recognition by analyzing patients’ head motion and eye movements (Sugihara et al., 2016). However, the small sample size could affect the interpretation of the results and further research should consolidate these findings.

Finally, we compared our studies to those included in the review of Pedroli et al. (2015) to verify whether researchers included a measure of the usability of VR-based USN assessment. Usability has been defined as the extent to which a product can be used by specified users to achieve specified goals with effectiveness, efficiency, and satisfaction in a specified context of use (Falcão and Marcelo, 2012; Medina et al. 2019). Therefore, it represents a relevant parameter that should be evaluated whenever a VR-based platform is tested or employed for clinical purposes. In line with Pedroli et al. (2015), the present review shows that usability is rarely evaluated when a VR-based platform is employed for the assessment of USN. Specifically, only two assessment studies included the System Usability Scale (SUS; Brooke, 1996) or an adapted version (Pallavicini et al., 2015a; Knobel et al., 2020).

#### Clinical considerations

From a clinical point of view, the hypothesis of USN/VSN as resulting from an attentional-perceptual bias appeared the most studied, and most authors addressed patients’ navigational impairments and collisions with contralesional static and moving obstacles, as well as their ability to timely initiate obstacle avoidance strategies (Aravind et al., 2015; Aravind and Lamontagne, 2017, 2018; Ogourtsova et al., 2018a). In this case, the virtual environment is capable of simulating life-like situations in which clinicians could safely observe patients’ behavioral responses and impairments. Several authors seemingly agree on considering USN/VSN as an attentional-perceptual deficit, with consequences in terms of properly detecting static and moving objects. Results showed that VSN attentional-perceptual bias is more prominent when patients are asked to simultaneously perform a locomotor and an increasingly more complex cognitive task, thus creating a dual-task interference. Furthermore, VSN patients tend to spend more time orienting their head toward the ipsilesional side, where they prefer to orient their attention (Aravind et al., 2015; Aravind and Lamontagne, 2017, 2018). Together, these findings could contribute to explaining patients’ behavior and VSN detrimental effect on their ability to timely initiate an avoidance strategy, leading to a higher collision rate with contralesional static and dynamic obstacles. Ogourtsova and colleagues (2018a, 2018b) also confirmed that USN patients manifest specific deficits both in spatial navigation and object detection as well as an impaired ability to update representation during navigation, which manifests with patients’ impaired ability to detect and adapt to a shifting target. Moreover, USN patients show significantly longer detection times and their performances worsened as a function of USN increased severity (Ogourtsova et al., 2018b).

Overall, the papers included in the present review seem to point in the direction of USN as an attentional-perceptual deficit, and the VR-based assessment seems increasingly more focused on considering patients’ actual behavior in life-like situations instead of cognitive functions isolated from the global functioning. This would allow to refine the assessment procedure and to detect even milder forms of USN, considering for instance how patients perceive and explore the environment (Kim et al., 2021). Despite the need for amelioration in terms of ecologicity, the environments and the technologies employed have allowed a refined assessment and provide data unobtainable otherwise, such as saccadic eye movements or reaction times.

### Technological advancements in USN rehabilitation

#### Methodological considerations

The search strategy performed, following the guidelines of Pedroli et al. (2015), lead to the selection of thirteen studies of VR-based USN rehabilitation (Kim et al., 2015; Faria et al., 2016; Fordell et al., 2016; Glize et al., 2017; Yasuda et al., 2017; Tobler-Ammann et al., 2017a, 2017b; Ekman et al., 2018; Wahlin et al., 2019; De Luca et al., 2019; Cogné et al. 2020; Choi et al., 2021; Huygelier et al., 2020). From a methodological perspective, six studies did not employ a control group (Kim et al., 2015; Fordell et al., 2016; Yasuda et al., 2017; Tobler-Ammann et al., 2017a; Ekman et al., 2018; Wåhlin et al., 2019), whereas only five studies either considered a control group of patients and/or healthy controls (Faria et al., 2016; Glize et al., 2017; Choi et al., 2021; Cogné et al. 2020; Huygelier et al., 2020) or compared patients’ experience to therapists’ (Tobler-Ammann et al., 2017b). Two studies were RCTs (Choi et al. 2021; Cogné et al. 2020) and two studies were a single case (De Luca et al., 2019; Yasuda et al., 2018).

Nine studies employed a non-immersive setting that consisted of a monitor, virtual environments, 3D vision glasses, a 3D robotic pen to perform tasks and provide feedback or a numeric keyboard, exergames, prism adaptation (Faria et al., 2016; Fordell et al., 2016; Ekman et al., 2018; Wahlin et al., 2019; Tobler-Ammann, 2017a, 2017b; Glize et al., 2017; De Luca et al., 2019; Cogné et al. 2020). Five studies employed a fully immersive setting that consisted of HMDs and motion-tracking devices (Kim et al., 2015; Yasuda et al. 2017, 2018; Choi et al. 2021; Huygelier et al., 2020).

Considerations regarding the ecological validity were extended to rehabilitation studies as well. Therefore, the present paper also examined how ecological were the virtual environments used for USN rehabilitation, compared to the studies considered by the paper of Pedroli et al. (2015). In the present review, some of the non-immersive devices recreated an ecological scenario that presented patients with realistic environments and requests like those they could face in the real world. Among these, a virtual supermarket where patients had to purchase items (Glize et al., 2017), or a virtual city in which patients could navigate with or without visual and auditory cues (Faria et al., 2016; Cogné et al. 2020). Other non-immersive devices included exergames designed to simulate increasingly difficult real-world tasks, (Tobler-Ammann et al., 2017a, 2017b). However, this setup could be considered only partially ecologic because, despite the tasks resembling daily-life situations, patients performed them in a seated position. One study provided life-like scenarios such as nature environments (De Luca et al., 2019) and seemed only partially ecological, whereas three other studies displayed three-dimensional figures that can be picked from shelves and moved from the left to the right side of the screen (Fordell et al., 2016; Ekman et al., 2018; Wahlin et al. 2019). This latter paradigm seems the least ecological, providing patients with geometrical and abstract stimuli. With respect to fully immersive devices, one study was particularly ecological in displaying multiple natural environments (a garden, a lake, and a forest) with different lighting (daytime, evening, or nighttime; Huygelier et al., 2020). Two studies exploited the same virtual room which presented patients with a desk placed in front of them (Yasuda et al. 2017, 2018). Patients were also capable of moving a virtual hand, that followed their actual movements, to reach for the object placed on the desk. The virtual room also displayed several objects on the far wall and could be considered sufficiently ecological. Another study presented a digitalized version of the line bisection task and could be considered the least ecological (Kim et al., 2015), as well as the digital practice setting (Choi et al. 2021). Therefore, the present review shows that virtual environments are becoming gradually more ecological, despite some of them keep asking patients to perform actions without physical interaction with the space (e.g., using a joystick or from a seated position) or present them with abstract/geometrical stimuli, which could be replaced with more realistic ones.

With respect to usability, the present review is consistent with the findings of Pedroli et al. (2015) showing that usability is rarely evaluated in rehabilitation studies as well. Among the studies employing non-immersive VR devices, only two studies assessed usability by means of the SUS (Faria et al., 2016) or evaluated perceived-user friendliness, attitude toward using the exergames, and their intention to use them in the future (Tobler-Ammann et al., 2017b). Only one study using fully immersive VR devices evaluated usability (and cybersickness) by means of a User Experience Scale (Huygelier et al., 2020).

#### Clinical considerations

From a clinical point of view, the attentional impairments involving the ventral and dorsal attentional networks and rightward attentional biases received the greatest attention, bolstering the hypothesis of USN as an attentional-perceptual deficit. Several rehabilitation studies focused on this cognitive function and RehAtt® was the most common program employed (Fordell et al., 2016). This VR-based training was created to act on both the ventral (VAN) and dorsal (DAN) attentional cerebral networks involved and possibly impaired in neglect. The RehAtt® proved its efficacy in enhancing patients’ spatial attentional performances; moreover, patients were able to transfer the improvements achieved following this rehabilitation training in daily life as well, and the same improvements lasted up to 6 months (Fordell et al., 2016). Further studies (Ekman et al., 2018; Wahlin et al. 2019) provided supporting fMRI data: cerebral changes induced by RehAtt® training are not limited to VAN and DAN but extend to the frontal eye field and the prefrontal regions associated with guiding attention and goal-directed behaviors (Ekman et al., 2018). Furthermore, fMRI showed an increased DAN inter-hemispheric functional connectivity and intrinsic neural communication (Wahlin et al. 2019). However promising, these results should be considered carefully, due to the limited sample size and the lack of a control group (Ekman et al., 2018; Wahlin et al. 2019).

As previously mentioned, the classical rehabilitation methods for USN include bottom-up interventions employing external instruments to manipulate patients’ sensory surroundings. Among these methods, prism adaptation (PA; Tsirlin et al., 2009; Jacquin-Courtois et al., 2010, 2013; Glize et al., 2019; Liu et al., 2019) and optokinetic stimulation (OKS; Pizzamiglio et al., 1990; Robertson et al., 1998; Moon et al., 2006) have received increasingly more attention over the years. In this paper, we present evidence of a virtual version of traditional USN rehabilitation: on one hand, PA was integrated in a VR task as a rehabilitation method, to decrease patients’ rightward attentional bias (Glize et al., 2017; Cogné et al. 2020). Results showed that PA improves patients' attentional resources and decreases their attentional bias. PA also seemingly exerts a positive effect on topographic and semantic information as well, possibly expanding to the recall of semantic knowledge (Glize et al., 2017). Moreover, the implementation of auditory cues seems to exert a positive effect on the navigational abilities of patients with visual and auditory neglect, even after a single PA exposure (Cogné et al., 2020). This suggests the feasibility of integrating PA and virtual environment within the rehabilitation of neglect and future studies could further explore this possibility. The employment of PA in both assessment and rehabilitation of neglect has thus shown promising preliminary results which should be deepened by future research. On the other hand, the combination of optokinetic stimulation (OKS) and HMD as rehabilitation for hemispatial neglect appeared a promising solution in helping patients see peripheral objects in their central visual field, which could also lead to neural reorganization and the reduction of neglect symptoms (Kim et al., 2015). The present work shows a positive effect of using non-immersive and fully immersive technologies in eliciting OKS (Kim et al., 2015) and exposing patients to prisms, which have proved effective after a single session (Glize et al., 2017; Cogné et al. 2020). However, the paucity of results for these integrated methods does not allow to draw solid conclusions, and further research should consolidate these preliminary findings with greater sample sizes.

Two studies focused on near and far space neglect in USN patients, first as a pilot study (Yasuda et al. 2017) and then as a single-case study (Yasuda et al., 2018). The VR-based rehabilitation employed in the first study appeared to exert a positive immediate impact on far space neglect, whereas patients apparently did not benefit from this training for the near-space (Yasuda et al. 2017). The second study showed a marked improvement on the patient’s neuropsychological measures following the same VR-based rehabilitation protocol, despite no changes were observed in the assessment of the patient’s performance of daily life activities (Yasuda et al., 2018). Once again, these results should be carefully considered given the lack of a control group and the methodological issues related to a single-case study.

Exergames also resulted in a safe and feasible complementary rehabilitation for visuospatial neglect (Tobler-Ammann et al., 2017a, 2017b), with positive group improvements in both cognitive and spatial exploration skills. This methodology appears a promising solution for promoting at-home rehabilitation as well. However, despite patients’ initial positive attitude toward exergames, they ended up finding them childish or boring or could not understand their purpose, thus preferring conventional therapy (Tobler-Ammann et al., 2017b). A similar result was obtained by therapists, which reported a more negative attitude and reservations on exergames’ therapeutic potential, with no intentions to use them in the future (Tobler-Ammann et al., 2017b).

Finally, only one single-case study employed the BTS-NIRVANA rehabilitation method, combined with standard cognitive training (De Luca et al., 2019). The study showed a promising correlation between an increase in P300 wave amplitude and improved cognitive functioning as a result of the S-IVT training. However, these results and their persistence overtime should be taken carefully due to the reduced sample size (De Luca et al., 2019).

The effectiveness of VR in the field of rehabilitation could be related to the feeling of presence induces by the most immersive VR devices. As previously mentioned, the sense of presence is closely related to the concept of “body matrix”, i.e., a multisensory, supramodal representation of the body and the (peripersonal) space around it. It integrates the many bodily representations, such as the sentient body, the spatial body, or the active body (Moseley et al., 2012; Riva 2018). The contents of the body matrix are adjusted depending on the (dis)agreement between the perceived sensory activity and the activity predicted based on the integration of different representations of the intentions of the self (Riva, 2008, 2018; Talsma, 2015). The predictive coding model suggests that the body matrix has a crucial role in minimizing the average of “surprise” and disparity between both different bodily representations and individuals’ intentions and effects of enacting them. The “surprise” is a free-energy condition, which all biological systems tend to minimize for survival purposes (Friston and Stephan, 2007; Friston et al., 2010; Riva, 2018). Therefore, the human brain should learn to model and predict the incoming sensory inputs, in order to minimize the number of free-energy states of surprise across the different bodily representations. This could be achieved by creating a high sense of presence in the body matrix, which allows locating the individual in the peripersonal space surrounding it (Riva 2008, Riva et al. 2018b). This spontaneous brain activity resembles what is achieved within the VR environment, which creates the sense of presence by integrating the input data collected via the trackers (sensing the user’s position and orientation) and a real-time update of the virtual environment created (Riva et al., 2018). VR thus exploits the simulation to subsequently predict the sensory consequences of the user’s movements and restore an expected sensory input (Riva et al., 2018). Following the theoretical framework of the embodied simulation, VR and the brain seemingly share their ability to employ simulations to predict the sensory consequences of the individual’s movements and provide an expected sensory output; this could account for VR’s effectiveness to improve health and well-being (Riva 2008, Riva et al., 2018b).

Overall, the papers included in the present review provide encouraging and positive results on the effects of VR-based rehabilitation of the navigational impairments and the attentional-perceptual biases of USN patients, observable by means of functional neuroimaging as well. These positive effects could be achieved with either non-immersive and fully immersive devices that display either newly developed interventions or a digitalized version of traditional rehabilitation paradigms, such as PA and OKS.

## Conclusion

The present review aims at updating the work of Pedroli et al. (2015) and provides an overview of the technological advancements in the VR-based assessment and rehabilitation of USN over the past six years. This section will briefly compare the conclusions drawn by Pedroli et al. with the results emerging from the present paper.

The paper of Pedroli et al. (2015) highlighted multiple strengths and challenges related to the use of VR in clinical contexts: VR tools are ergonomic and well-adaptable to patients’ needs and conditions, particularly if they have paretic limbs or need a wheelchair for locomotion. The present paper is in line with the work of Pedroli et al. (2015) and shows that both assessment and rehabilitation can benefit from VR-based platforms that easily adapt to patients’ needs and impairments without losing their properties.

Secondly, the paper of Pedroli et al. (2015) outlined that clinicians could lack technical skills and need to collaborate with software developers in order to use VR systems properly. The authors emphasized the need for specific training addressed at clinicians, as well as the development of intuitive VR applications. To date, the use of VR technologies in a clinical setting (e.g., a hospital) continues to entail some issues, such as the need for specific training for their correct use and an appropriate setting. The present review agrees with the work of Pedroli and colleagues: the need for specific training persists particularly for the most advanced VR platforms (e.g., the CAVE), whereas other devices (e.g., HMDs, tablets) require only a minimal technological background, at least for clinicians. Collaboration with software developers is still strongly recommended. Moreover, patients that would autonomously use portable devices (e.g., tablets), particularly for at-home rehabilitation on elderlies, should still receive training on how to use them safely. To solve this issue, clinicians could provide availability in case patients need assistance while using portable devices.

Thirdly, the paper highlighted that the considerable cost of VR technology could hamper its implementation in clinical settings and suggested that the development of portable devices at reasonable prices would represent a viable solution for telemedicine (Pedroli et al., 2015). The most immersive VR devices are quite expensive and space-consuming but, most importantly, they cannot be transferred to the patients’ house allowing them to prosecute at-home rehabilitation, which could consolidate the benefits of the intervention during the hospitalization period (Pallavicini et al., 2015a). In 2015, the acquisition of Oculus Rift operated by Facebook has allowed the widespread use of these devices, which became increasingly more affordable and available not only for the general public but also for healthcare services. Indeed, a limited budget could hinder the employment of more advanced immersive and semi-immersive technologies for research purposes and for clinical practice within a hospital and could be impossible for the private practitioner to afford them. A plausible and accessible compromise would be the use of Google Cardboards, for instance: despite the limited quality, compared to other technologies, this platform could be helpful in the dissemination of VR devices within the clinical context as well. The present work shows an increase in the use of these devices as a tool for both assessment and rehabilitation, their usefulness, and how they contribute to promoting telemedicine. The use of portable VR devices after patients’ dismissal allows them to prosecute their rehabilitation and consolidate its beneficial effects at home as well, in a more familiar setting. This enables clinicians to monitor patients’ conditions over time, enhances patients’ engagement and treatment adherence, and, most importantly, promotes an autonomous aging-in-place which is particularly relevant in this historical moment, particularly following the Covid-19 pandemic outbreak and its after-effects (Holden, 2005; Lange et al., 2010; Stones and Gullifer, 2014; de Rooij et al., 2016; Kim et al., 2017; Serino et al., 2017a, 2017b; Mugueta-Aguinaga and Garcia-Zapirain 2017; Kidd et al., 2019)

Pedroli and colleagues (2015) outlined that, despite VR raising patients’ interest and participation, very few studies used these new technologies in the field of USN assessment and rehabilitation, and those studies showed several methodological flaws. Specifically, only a limited number of research compared VR to conventional methods or a control group, and the limited sample size would limit external validity and generalization of the results (Pedroli et al., 2015). Furthermore, the authors highlighted that, despite being one of the most effective rehabilitation interventions, the prisms technique was one of the least used (Pedroli et al., 2015). The results of this 6-year update show that several transitions and major steps forward have been made in the field of VR-based assessment and rehabilitation of USN. Whether the first major transition has allowed to digitalize the paper-and-pencil tools and develop computerized forms of neuropsychological tests and rehabilitation, the second major transition has allowed stepping from a less realistic presentation of the stimuli to a more ecological and immersive simulation of lifelike situations, that allow a more precise and thorough examination of patients’ functioning. This has been fostered by the widespread dissemination of technologies such as the HMDs, which have become gradually more available and affordable and allow a refined detection of saccadic eye movements, eye rotations, and head position (Huygelier et al., 2020). However, this 6-year update also endorses the work of Pedroli et al. (2015) in highlighting frequent methodological issues and limitations that should be addressed by future researchers, such as the lack of control group, randomization, or reduced sample sizes, which could jeopardize the results or limit the generalizability of the outcomes. Several authors of the papers included in the present work outline the need for more research to establish the feasibility and the efficacy of VR-based assessment and rehabilitation for USN, as well as (Faria et al., 2016; Choi et al. 2021; Huygelier et al., 2020). Thus, we recommend future research to enhance methodological rigor by means of RCTs, greater sample sizes, and adequate follow-up periods as well, in order to monitor the persistence of the improvements following a rehabilitation. Moreover, despite the greater use of technologies, a proper evaluation of the usability of VR devices is currently lacking. This limitation was found both in the papers presented by Pedroli et al. (2015) and in the research considered in the present review as well. Therefore, we strongly recommend future studies to evaluate usability carefully and systematically, given the wide range of scales and questionnaires available (Falcão and Marcelo, 2012; Medina et al. 2019).

Finally, the paper of Pedroli et al. suggested that future studies would allow a more fluid interaction within the environment by means of motion-tracking systems (e.g., wired gloves, Kinect, Vicon). The present work outlines that the greater diffusion of non-immersive and fully immersive devices, integrating traditional assessment and rehabilitation methods, provides a more ecological context and exploits motion-tracking systems. Furthermore, the present review has considered whether the virtual environments and the stimuli employed in the assessment and rehabilitation of USN could be considered ecological, compared to the papers considered in the previous work of Pedroli et al. (2015). Despite, over the years, research has employed more ecological scenarios of tasks that patients had to perform, some of the virtual environments or the stimuli provided are still scarcely realistic and too abstract. Therefore, we strongly recommend future studies to further improve the ecological validity of VR-based USN assessment and rehabilitation, by means of like-like scenarios that would allow considering patients’ actual functioning and impairments in performing daily tasks. Finally, future studies should consolidate the integration of VR with other technologies, such as transcranial magnetic stimulation (TMS; Cassani et al., 2020; Mancuso et al., 2020) and eye-tracking (Clay et al. 2019; Davis 2021) for the assessment and rehabilitation of USN patients.

Elisa Pedroli created the original methodology of the study. Literature search results found by the first author (Silvia Cavedoni) were shared with the review co-authors (Pietro Cipresso, Valentina Mancuso) for individual selection of papers in order to reduce bias, and disagreements were resolved through consensus. Silvia Cavedoni wrote the first draft of the manuscript and all authors critically reviewed previous versions. All authors read and approved the final manuscript. All authors agree to be accountable for all aspects of the work in ensuring that questions related to the accuracy or integrity of any part of the work are appropriately investigated and resolved.
